# Mutations of Human *NARS2*, Encoding the Mitochondrial Asparaginyl-tRNA Synthetase, Cause Nonsyndromic Deafness and Leigh Syndrome

**DOI:** 10.1371/journal.pgen.1005097

**Published:** 2015-03-25

**Authors:** Mariella Simon, Elodie M. Richard, Xinjian Wang, Mohsin Shahzad, Vincent H. Huang, Tanveer A. Qaiser, Prasanth Potluri, Sarah E. Mahl, Antonio Davila, Sabiha Nazli, Saege Hancock, Margret Yu, Jay Gargus, Richard Chang, Nada Al-sheqaih, William G. Newman, Jose Abdenur, Arnold Starr, Rashmi Hegde, Thomas Dorn, Anke Busch, Eddie Park, Jie Wu, Hagen Schwenzer, Adrian Flierl, Catherine Florentz, Marie Sissler, Shaheen N. Khan, Ronghua Li, Min-Xin Guan, Thomas B. Friedman, Doris K. Wu, Vincent Procaccio, Sheikh Riazuddin, Douglas C. Wallace, Zubair M. Ahmed, Taosheng Huang, Saima Riazuddin

**Affiliations:** 1 Department of Developmental and Cellular Biology, School of Biological Sciences, University of California, Irvine, Irvine, California, United States of America; 2 CHOC Childrens’, Division of Metabolics, Orange, California, United States of America; 3 Department of Otorhinolaryngology Head & Neck Surgery, School of Medicine, University of Maryland, Baltimore, Maryland, United States of America; 4 Division of Human Genetics, Cincinnati Children’s Hospital Medical Center, Cincinnati, Ohio, United States of America; 5 National Center for Excellence in Molecular Biology, University of the Punjab, Lahore, Pakistan; 6 Center for Mitochondrial and Epigenomic Medicine, Children’s Hospital of Philadelphia and Department of Pathology and Laboratory Medicine, University of Pennsylvania, Philadelphia, Pennsylvania, United States of America; 7 Division of Pediatric Otolaryngology Head & Neck Surgery, Cincinnati Children’s Hospital Medical Center, Cincinnati, Ohio, United States of America; 8 Smilow Center for Translational Research, University of Pennsylvania, Philadelphia, Pennsylvania, United States of America; 9 Trovagene, San Diego, California, United States of America; 10 Marshall B Ketchum University, Fullerton, California, United States of America; 11 Department of Physiology and Biophysics, University of California, Irvine, Irvine, California, United States of America; 12 Manchester Centre for Genomic Medicine, University of Manchester and Central Manchester University Hospitals NHS Foundation Trust, Manchester Academic Health Sciences Centre (MAHSC), Manchester, United Kingdom; 13 Department of Neurology and Neurobiology, University of California, Irvine, Irvine, California, United States of America; 14 Division of Developmental Biology, Cincinnati Children’s Hospital Medical Center, Cincinnati, Ohio, United States of America; 15 Swiss Epilepsy Center, Zurich, Switzerland; 16 Institute of Molecular Biology, Mainz, Germany; 17 Institute for Genomics and Bioinformatics, University of California, Irvine, Irvine, California, United States of America; 18 Architecture et Réactivité de l’ARN, CNRS, University of Strasbourg, IBMC, Strasbourg, France; 19 Parkinson’s Institute and Clinical Center, Sunnyvale, California, United States of America; 20 Laboratory of Molecular Genetics, National Institute on Deafness and Other Communication Disorders, National Institutes of Health, Bethesda, Maryland, United States of America; 21 Section on Sensory Cell Regeneration and Development, National Institute on Deafness and Other Communication Disorders, National Institutes of Health, Bethesda, Maryland, United States of America; 22 Biochemistry and Genetics Department, UMR CNRS 6214–INSERM U1083, CHU Angers, Angers, France; 23 Jinnah Hospital Complex, Allama Iqbal Medical College, University of Health Sciences, Lahore, Pakistan; 24 University of Lahore, Lahore, Pakistan; 25 Shaheed Zulfiqar Ali Bhutto Medical University, Islamabad, Pakistan; Tel Aviv University, ISRAEL

## Abstract

Here we demonstrate association of variants in the mitochondrial asparaginyl-tRNA synthetase *NARS2* with human hearing loss and Leigh syndrome. A homozygous missense mutation ([c.637G>T; p.Val213Phe]) is the underlying cause of nonsyndromic hearing loss (DFNB94) and compound heterozygous mutations ([c.969T>A; p.Tyr323*] + [c.1142A>G; p.Asn381Ser]) result in mitochondrial respiratory chain deficiency and Leigh syndrome, which is a neurodegenerative disease characterized by symmetric, bilateral lesions in the basal ganglia, thalamus, and brain stem. The severity of the genetic lesions and their effects on NARS2 protein structure cosegregate with the phenotype. A hypothetical truncated NARS2 protein, secondary to the Leigh syndrome mutation p.Tyr323* is not detectable and p.Asn381Ser further decreases NARS2 protein levels in patient fibroblasts. p.Asn381Ser also disrupts dimerization of NARS2, while the hearing loss p.Val213Phe variant has no effect on NARS2 oligomerization. Additionally we demonstrate decreased steady-state levels of mt-tRNA^Asn^ in fibroblasts from the Leigh syndrome patients. In these cells we show that a decrease in oxygen consumption rates (OCR) and electron transport chain (ETC) activity can be rescued by overexpression of wild type *NARS2*. However, overexpression of the hearing loss associated p.Val213Phe mutant protein in these fibroblasts cannot complement the OCR and ETC defects. Our findings establish lesions in *NARS2* as a new cause for nonsyndromic hearing loss and Leigh syndrome.

## Author Summary

Mitochondrial respiratory chain (MRC) disease represents a large and heterogeneous group of energy deficiency disorders. Here we report three mutations in NARS2, a mitochondrial asparaginyl-tRNA synthetase, associated with non-syndromic hearing loss (NSHL) and Leigh syndrome in two independent families. Located in the predicted catalytic domain of the protein, missense mutation p.(Val213Phe) results in NSHL (DFNB94) while compound heterozygous mutation (p.Tyr323*; p.Asn381Ser) is leading to Leigh syndrome with auditory neuropathy. *In viv*o analysis deemed p.Tyr323* mutant protein to be unstable. Co-immunoprecipitation assays show that p.Asn381Ser mutant disrupts the dimerization ability of NARS2. Leigh syndrome patient fibroblasts exhibit a decreased steady-state level of mt-tRNA^Asn^. In addition, in these cells, the mitochondrial respiratory chain is deficient, including significantly decreased oxygen consumption rates and electron transport chain activities. These functions can be partially restored with over-expression of wild-type NARS2 but not with p.Val213Phe mutant protein. Our study provides new insights into the genes that are necessary for the function of brain and inner ear sensory cells in humans.

## Introduction

Mitochondrial respiratory chain (MRC) disease represents a large and heterogeneous group of energy deficiency disorders [1]. A significant percentage of MRC disorders is caused by both nuclear and mitochondrial encoded genetic variants that impact molecules of the mitochondrial protein synthesis machinery [2]. Among these genes, those coding for the mitochondrial aminoacyl-tRNA synthetases (mt-aaRSs) have emerged as being frequently associated with human disease [3]. The primary function of mt-aaRSs is to charge mitochondrial tRNA (mt-tRNA) molecules with their cognate amino acids [4]. Scheper and co-authors first linked mutations in *DARS2*, encoding the mitochondrial aspartyl-tRNA synthetase, to leukoencephalopathy with brain stem and spinal cord involvement and lactate elevation in brain (LBSL; MIM: 611105) [5]. Since then, mutations of 14 other mt-aaRSs have been associated with mitochondrial disease [6,7], including *NARS2*, which has recently been linked to intellectual disability, epilepsy and myopathy [8]. The significant tissue specificity and phenotypic heterogeneity of mutated mt-aaRSs was unexpected, considering that a deficiency in these ubiquitously expressed enzymes should affect all tissue types [9]. The delineation of the underlying cellular mechanisms is subject to intensive investigation for cytoplasmic as well as mitochondrial aaRS [10,11].

Mutations of five (mt) aaRS genes cause syndromic forms of deafness, including Perrault syndrome (*LARS2*, *HARS2*), Charcot Marie Tooth disease type 2N (*AARS*) [12] and pontocerebellar hypoplasia type 6 (*RARS2*) [13]. Homozygous mutations in *KARS*, an aaRS which functions in the cytoplasm as well as the mitochondria, have been shown to cause nonsyndromic hearing impairment (DFNB89) [14]. The gene shows similar pleiotropism as it is demonstrated for *NARS2* in this study since in addition to DFNB89, compound heterozygous *KARS* mutations have been shown to cause Charcot Marie Tooth disease and developmental delay as well as severe infantile disease with microcephaly and white matter abnormalities, seizures and vision loss [12,15].

Mutations in *EARS2* and *FARS2* have been shown to cause fatal epileptic mitochondrial encephalopathy and/or Alpers syndrome [16–18] and mutations in *IARS2* have previously been associated with Leigh Syndrome or Leigh-like disease. Our report therefore adds *NARS2* to the list of mt-aaRS associated fatal epileptic mitochondrial encephalopathy and represents the second Leigh syndrome associated mt-aaRS [7]. Leigh syndrome is a neurodegenerative disease caused by mitochondrial dysfunction resulting in symmetric, bilateral lesions in the basal ganglia, thalamus, and brain stem [19,20]. Leigh syndrome is the most common clinical finding associated with mitochondrial disease of childhood and displays significant genetic heterogeneity [20,21]. To date there are over 60 genes associated with Leigh syndrome, and a large proportion is caused by defects in molecules involved in the mitochondrial translational machinery [20]. In two families, we report phenotypic variability associated with different mutations of the same mt-aaRSs, NARS2. One family is segregating nonsyndromic hearing loss (DFNB94) and another with Leigh syndrome.

## Results

### Clinical findings

#### Family LS06

**Fig 1:**
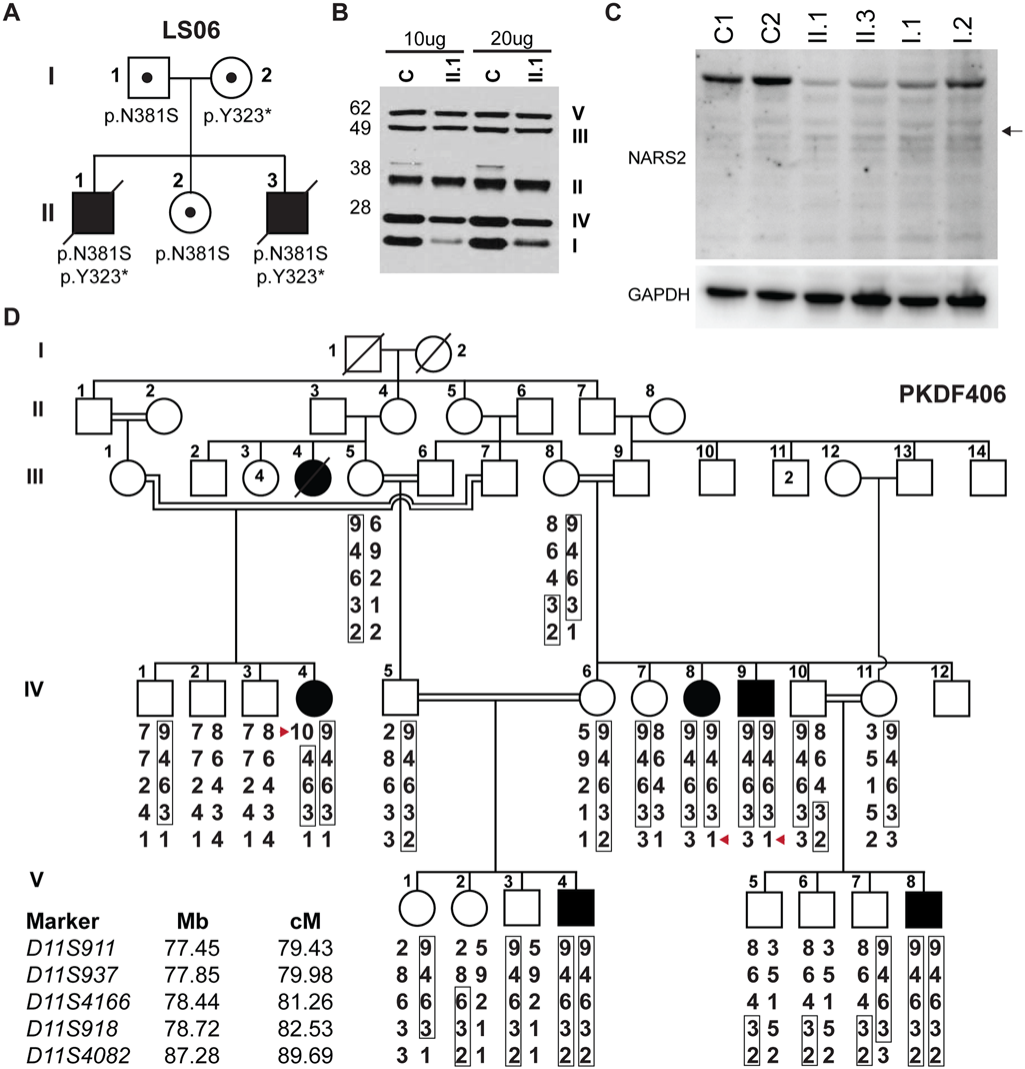
*NARS2* mutations identified in two unrelated families. (A) Pedigree of the LS06 family. Filled symbols represent affected individuals and small circles represent carrier individual. The pedigree shows autosomal recessive inheritance of compound heterozygous NARS2 variants [c.969T>A; p.Tyr323*] and [c.1142A>G; p.Asn381Ser]. (B) SDS PAGE and Western blot of control and patient II.1 muscle homogenates (10μg and 20μg of protein), samples were probed for mitochondrial respiratory chain complexes via MitoProfile total OXPHOS human WB antibody cocktail. The result showed significantly decreased amounts of mitochondrial respiratory complex I and IV. (C) SDS PAGE and Western blot of fibroblast lysates from both affected probands (II.1, II.3), their parents (I.1, I.2) and controls using anti-NARS2 antibody and anti-GAPDH antibody as loading control. The expected position of a truncated NARS2 protein product (Δ154aa) stemming from the p.Tyr323* allele is indicated with a black arrow. (D) Pedigree of the PKDF406 family. Filled symbols represent affected individuals, and a double horizontal line represents a consanguineous marriage. Alleles forming the risk haplotypes are boxed. The short tandem repeat (STR) markers, their relative map positions (Mb) according to UCSC Genome Bioinformatics build GRCh37 (hg19), and their genetic positions (cM) based on the Marshfield genetic map are shown next to the pedigree. A haplotype analysis revealed a linkage region delimited by a proximal meiotic recombination at marker D11S911 in individual IV:4 (arrowhead) and distal recombination at marker D11S4082 in individuals IV:8 and IV:9 (arrowhead).

Subject II.1, from family LS06, (Fig. 1A) was born to unrelated healthy Caucasian parents without contributory family history. He was considered normal at birth but then failed the post-natal hearing screen. Follow up testing at 1 month of age showed absent Auditory Brainstem Response (ABR) with preserved cochlear microphonics, diagnostic of bilateral auditory neuropathy. Pure tone otoacoustic emissions (OAEs) testing at 11 weeks showed absent transient evoked emissions. Early developmental milestones were normal. Myoclonic movements started at 3 months of age, rapidly worsened, eventually involving all four extremities and complex partial seizures were recorded. A CT scan and MRI of the head were normal. Extensive laboratory studies for metabolic disease showed abnormal urine organic acids with elevations in multiple TCA cycle metabolites (S1 Table). CSF lactate was elevated at 3.9 mmol/L (Normal <2.0 mmol/L) and 5.4 mmol/L by 5 months of age, while plasma lactate was normal.

Follow up MRI showed multiple areas of hyperintensive T2-weighted and Fluid-attenuated inversion recovery (FLAIR) signal within periventricular white matter and posterior corona radiata with extension into the posterior limbs of the internal capsule.There was also a hyperintensive signal in the thalami and dentate nuclei. Electroencephalography (EEG) was abnormal, consistent with status epilepticus. Anti-seizure medications were tried including Topamax, Dilantin and Klonopin, but were not effective. By 10 months of age, he had developed laryngomalacia with pharyngeal hypotonia, his condition progressed and he died of respiratory failure at 15 months of age. Post mortem examination of the brain showed cortical atrophy with laminar necrosis, atrophy of the corpus callosum, significant white matter oligodendroglial loss. Neuronal gray matter loss was widespread with gliosis. Multifocal prominent hypervascularity, as well as, symmetrical lesions in the brainstem and thalamus were characteristic of Leigh syndrome.

A muscle sample was obtained post-mortem within the acceptable time frame for preservation of mitochondrial enzyme activities. Histology, including mitochondrial stains and electron microscopy, was normal. ETC studies showed absent activity of NADH cytochrome C reductase (COI/III), indicating a block in electron flow from the membrane bound arm of mitochondrial complex I (COI) to mitochondrial complex III (COIII) and a milder reduction in complex IV (COIV) activity. Mitochondrial ferricyanide reductase activity probing the matrix arm of COI, which is thought to contain mainly nuclear encoded subunits, was normal (S2 Table).

Additionally SDS PAGE and Western blot for muscle and fibroblast lysates from proband II.1 were performed with a standard protocol (29). Consistent with the ETC results, Western blot on muscle lysates showed significantly decreased levels for mitochondrial complex I (NDUFB8–18kD) and moderate decrease for mitochondrial complex IV (MTCOII-22kD) (Fig. 1B). However, Western blot performed with anti-GRIM19 antibody for mitochondrial complex I (GRIM19 corresponds to the mitochondrial complex I subunit NDUFA13) in fibroblast lysates was normal (S1A Fig).

The family’s third child, a male who was born after an uneventful pregnancy via spontaneous vaginal delivery (II.3, Fig. 1A) also failed the new born hearing screen but was otherwise normal. ABR and OAE testing at 1 month of age showed comparable bilateral auditory neuropathy with normal middle ear function. He first presented with myoclonic movements of the right arm at three months of age, accompanied by lethargy and decreased feedings, which necessitated the placement of a gastrostomy tube. The MRI showed restricted diffusion in the left basal ganglia, and external capsule junction as well as the left frontal lobe in cortical distribution. EEG showed continuous left hemispheric focal seizures. Laboratory testing for organic acids revealed mild elevation in TCA cycle metabolites, fumaric, malic, and 2-keto-glutaric acids; suggestive of mitochondrial disease. Plasma amino acids, as well as, lactic acid in blood were within normal range. A muscle biopsy at three months of age was normal for coenzyme Q levels and histology, while ETC studies (CIDEM) showed complex I/III deficiencies similar to his deceased brother. Individual II.3 passed away at 6 months of age. Autopsy also showed severe encephalopathy and prominent basal ganglia involvement consistent with Leigh syndrome. There were fewer vaso-proliferative lesions than in proband II.1. The striate cortex showed severe degeneration, which correlated with the patient’s cortical blindness. Microscopically there was widespread gliosis and prominent diffuse metabolic astrocytosis.

#### Family PKDF406

We ascertained family PKDF406 from Punjab province of Pakistan. The pedigree suggested that deafness was segregating in this family as an autosomal recessive trait (Fig. 1D). Pure-tone audiometric evaluations of the affected individuals from the PKDF406 family revealed pre-lingual, profound, bilateral sensorineural hearing loss (HL) (Table 1, S2 Fig). We found no evidence of co-segregation of vestibular dysfunction, retinitis pigmentosa or an obvious cognitive disability with HL in the PKDF406 family (Table 1).

**Table 1:** Clinical findings of affected PKDF406 family members.

No.	Age	Sex	Hearing status	Retinitis pigmentosa	Vestibular function^a^	Hypotonia	Seizure history	Brain CT scan	Menstrual history
IV:4	40 y	F	Profound	No	Normal	No	None	ND	Menopause ^c^
IV:8	45 y	F	Profound	No	Normal	No	None	ND	Menopause ^c^
V:8	26 y	M	Severe to profound	No	Normal	No	None	Right maxillary sinusitis	NA
V:4	30 y	M	Profound	No	Positive^b^	No	None	Normal	NA

^a^Evaluated using Tandem gait and Romberg tests.

^b^Positive: fumbled during both tests.

NA: Not applicable; ND: Not determined.

^c^Had history of normal menstrual cycles before premature menopause

### Leigh syndrome and nonsyndromic hearing loss caused by mutations of *NARS2*

#### Leigh syndrome caused by mutations of *NARS2*

mtDNA studies for patient II.1 from family LS06, identified a heteroplasmic variant in the mitochondrial tRNA cysteine (mt-tRNA^Cys^) at position A5793G while all other studies were normal (S3 Fig). Segregation analysis showed the variant to be homoplasmic in the unaffected sister and could therefore not have been the underlying cause of the phenotype (S3B Fig). Since the variant could cause suboptimal translation of mtDNA encoded respiratory chain subunits we performed transmitochondrial cybrid studies. The variant did not affect mitochondrial complex I levels when introduced into a neutral nuclear background (S1 Fig). Therefore, to identify the disease-causing gene, we performed whole exome sequencing (WES) for probands II.1 and II.3 and their father (I.1). This analysis revealed compound heterozygous variants in the *NARS2* gene (NM_024678, MIM: 612803), encoding mitochondrial asparaginyl-tRNA synthetase (mt-AsnRS or NARS2), at positions [c.969T>A; p.Tyr323*] and [c.1142A>G; p. Asn381Ser] (Fig. 1A, S4 Fig). Carriers of these mutations were not found in the 1000 Genome Project or the NHLBI Exome Variant Server (EVS). The *NARS2* variants represent the only candidate gene, which could explain the proband’s phenotype (S3 Table).

#### Nonsyndromic hearing loss caused by mutation of *NARS2*

In family PKDF406, linkage analysis was undertaken using short tandem repeat (STR) markers for many of the reported recessive nonsyndromic deafness loci. PKDF406 family was found to be segregating deafness linked to markers for *DFNB2* (Fig. 1D) on chromosome 11q13.5 [22]. Previous studies have shown that mutant alleles of *MYO7A* are responsible for the DFNB2 phenotype in humans [23,24]. Using the NGS-based mutation screening test OtoSeq [25], we sequenced the affected individual IV:8 (Fig. 1D) from the PKDF406 family and did not find any pathogenic variants in all of the coding and non-coding exons of *MYO7A*. Furthermore, refined mapping and haplotype analyses using additional PKDF406 family members excluded the *MYO7A* gene from the linkage interval on chromosome 11q13.4-q14.1. Therefore, the HUGO nomenclature committee assigned the designation *DFNB94* to the locus defined by the PKDF406 family. A maximum two-point LOD score of 5.10 (θ = 0) was obtained for marker *D11S937*.

Next, genomic DNA from individual IV:8 of family PKDF406 was processed for WES. All the variants found in the WES data are summarized in S4 Table. Since there was significant evidence of linkage of deafness segregating in family PKDF406 to STR markers on chromosome 11q13.4-q14.1, we focused only on the variants present in the *DFNB94* linkage interval. We found a c.637G>T transversion variant in *NARS2*. Using Sanger sequencing (S4 Fig), we confirmed the segregation of the c.637G>T allele with hearing loss in the PKDF406 family. Sanger sequencing of all the coding and non-coding exons of *NARS2* (S4 Table) did not reveal any other changes besides c.637G>T in family PKDF406. No carrier of the c.637G>T mutation was found in the 500 ancestry-matched control chromosomes, the 1000 Genome Project or the NHLBI EVS database.

### *Nars2* expression in the brain and inner ear

**Fig 2:**
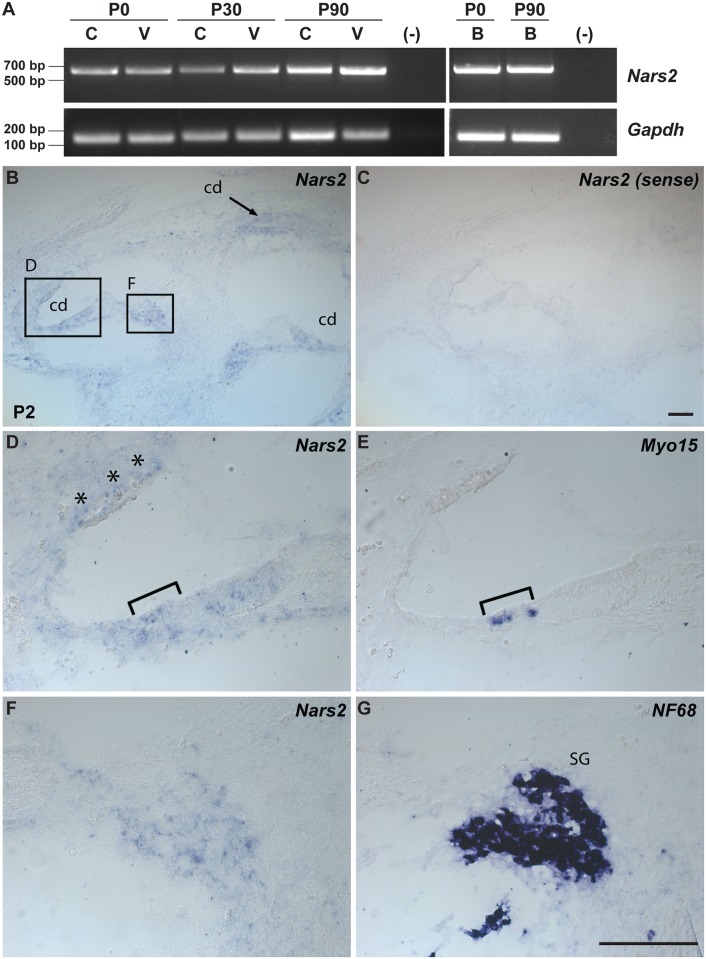
Expression of *Nars2* in mouse inner ear and brain. (A) RT-PCR analysis of *Nars2* expression in the C57Bl6/J mouse cochlear (C), vestibular (V) tissues and brain (B) at different developmental stages (P0, P30 and P90). *Gapdh* expression was used as an internal control. (B-G) Expression of *Nars2* in the P2 mouse cochlea is shown. Hybridization signals of *Nars2* antisense (B, D, F) and sense (control) probes (C) in mid-modiolar sections of P2 cochlea are shown. Positive signals were detected in the cochlear epithelium, including the region of the organ of Corti, (D) as indicated by the Myo15a-positive hair cells (E, bracket). Positive signals were also detected in the cells surrounding the cochlear duct (D, asterisks) and neurofilament-positive spiral ganglion (G, SG). B, C, E and G are 12 mm adjacent sections. Abbreviations: cd, cochlear duct, SG, spiral ganglion. The scale bar in C is 100 μm and applies to B and C. The scale bar in G is 100 μm and applies to D-G.

**Fig 3:**
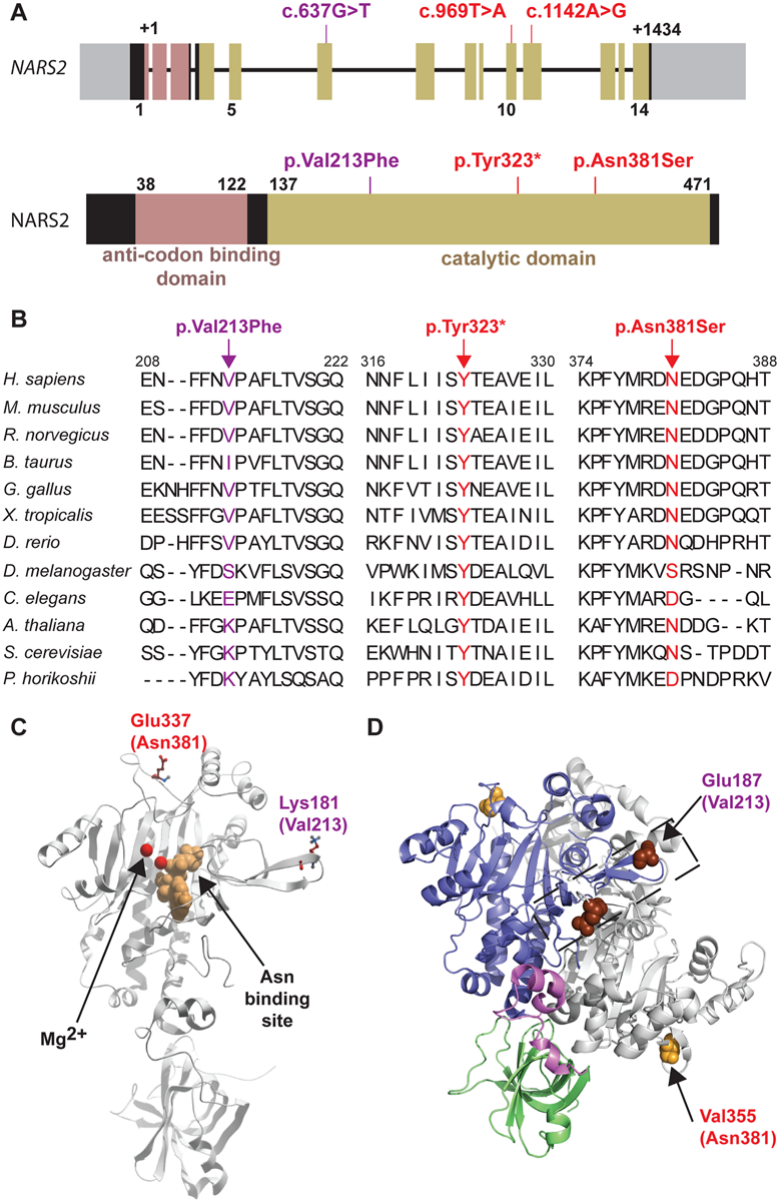
*NARS2* structure and molecular modeling. (A) Schematic representation of *NARS2* gene and predicted encoded protein product. Exons are represented with boxes. The anti-codon binding domain is shown in pink, and the catalytic domain is shown in beige. The c.637G>T, c.969T>A, c.1142A>G mutations are located in exons 6, 10 and 11, respectively, which are all coding for a part of the catalytic domain of the protein. (B) Protein sequence alignments show the evolutionary conservation of the mutated residues (arrows). (C) Mapping of homologs of the p.Val213Phe and p.Asn381Ser mutations in a 3D structure of NARS2. This 3D model of the NARS2 protein is based on the structure of *Pyrococcus horikoshii* AsnRS. The asparagine binding site is represented in beige; Mg^2+^ ions are shown in red. The human mutations p.Val213Phe and p.Asn381Ser were mapped on the *Pyrococcus horikoshii* molecule at position p.Lys181 and p.Glu337, respectively. (D) Dimeric representation of *Entamoeba histolytica* AsnRS (PDB: 3M4Q) using PyMol molecular graphics system. Anticodon binding domain is represented in green, hinge region in pink, catalytic core in blue and second monomer is shown in grey. The *E*. *histolytica* residue corresponding to human NARS2 p.Asn381 is p.Val355 and shown by an orange sphere. The *E*. *histolytica* residue corresponding to human p.Val213 is p.Glu197 and is displayed in dark brown.

*NARS2* is widely expressed in human and mouse tissues (UniGene, see Web Resources), including the brain, cochlear and vestibular systems (Fig. 2A, S5 Table). In mouse brain, *Nars2* is also broadly expressed with prominent *in situ* hybridization signals in regions such as the cortex, hippocampus, cerebellum and brain stem (Allen Brain Atlas; see Web Resources). A transcriptome analysis showed a 10-fold increase in *Nars2* mRNA in mouse cochlear and vestibular spiral ganglion cells at postnatal day 0 (P0) compared with sensory hair cells (SHIELD; see Web Resources). As there is no commercially available antibody for murine Nars2, we performed *in situ* hybridization studies to highlight the expression of *Nars2*. They revealed a broad *Nars2* expression pattern in the spiral ganglion (Fig. 2B, F), the cochlear duct including the organ of Corti (Fig. 2D, bracket), and some of the mesenchyme surrounding the duct (Fig. 3D, asterisks), at P2 (Fig. 2B, D, F). A *Nars2* sense probe was used as a negative control (Fig. 2C), and anti-sense probes for *Myo15a* and *NF68* (Fig. 2E, 2G) were used as positive controls for labeling sensory hair cells and spiral ganglion, respectively [26,27].

### Mutations located in exons encoding the predicted catalytic domain of NARS2

*NARS2* is composed of 14 exons that encode a protein of 477 amino acids (Fig. 3A). *NARS2* was first described in yeast [28], and belongs to the class II aminoacyl-tRNA synthetases, a classification based on three consensus sequence motifs in the catalytic subunit [29,30]. Generally, aaRS are comprised of an anticodon binding domain and a catalytic domain. Some aaRSs have an additional domain with editing functions to prevent the insertion of inappropriate amino acids during protein synthesis. However, InterProScan [31] and SWISS-MODEL [32] molecular modeling softwares predicted that NARS2 does not contain this additional domain (Fig. 3A). All mutations observed in our families are located in the predicted catalytic domain (Fig. 3A). The Leigh syndrome associated p.Tyr323* variant results in a premature termination codon and is therefore considered damaging. The stop codon occurs instead of the tyrosine residue which is conserved through yeast (Fig. 3B) and hypothetically results in a truncated protein of 323 amino acids. The second Leigh syndrome variant p.Asn381Ser substitutes Serine for a highly conserved Asparagine with a GERP score of 4.59 (Fig. 3B). This variant is predicted highly pathogenic by all ten interrogated pathogenicity prediction programs (S6 Table). The hearing loss p.Val213Phe variant is moderately conserved with a GERP score of 3.89. It is deemed highly damaging by 5 out of the 10 interrogated pathogenicity algorithms [33,34].

The 3D structure of human NARS2 has not been resolved. However, there is 30.5% identity and 50.1% similarity between NARS2 and *Pyrococcus horikoshii* AsnRS, which allowed us to use the crystal structure of *Pyrococcus horikoshii* AsnRS [35] to model the effect of the human missense mutations of NARS2. Human NARS2 p.Val213 residue corresponds to the p.Lys181 residue in *Pyrococcus horikoshii (* Fig. 3B-C). Molecular modeling data suggests that the p.Lys181 residue is present on the surface of the molecule (Fig. 3C). The substitution of this valine residue with phenylalanine in humans is predicted to create a sticky patch on the surface that could affect the protein-protein interactions of NARS2. Since a dimeric protein form has been described for mt-AsnRS of *Entamoeba histolytica* (Protein Data Bank access # 3M4Q), we used it as backbone for *in silico* modeling. The p.Glu187 and p.Val355 of *Entamoeba histolytica* mt-AsnRS correspond to p.Val231 and p.Asn381 residues in human NARS2 (Fig. 3D). Molecular modeling data suggest that p.Asn381 is not directly involved in the dimer interphase. However, the variant could affect the dimerization by propagation of a structural perturbation. Conversely, the p.Val213 residue is located within the dimer interphase. We therefore functionally interrogated a potential NARS2 oligomerization defect.

### p.Asn381Ser-NARS2 does not dimerize with wild type NARS2

**Fig 4:**
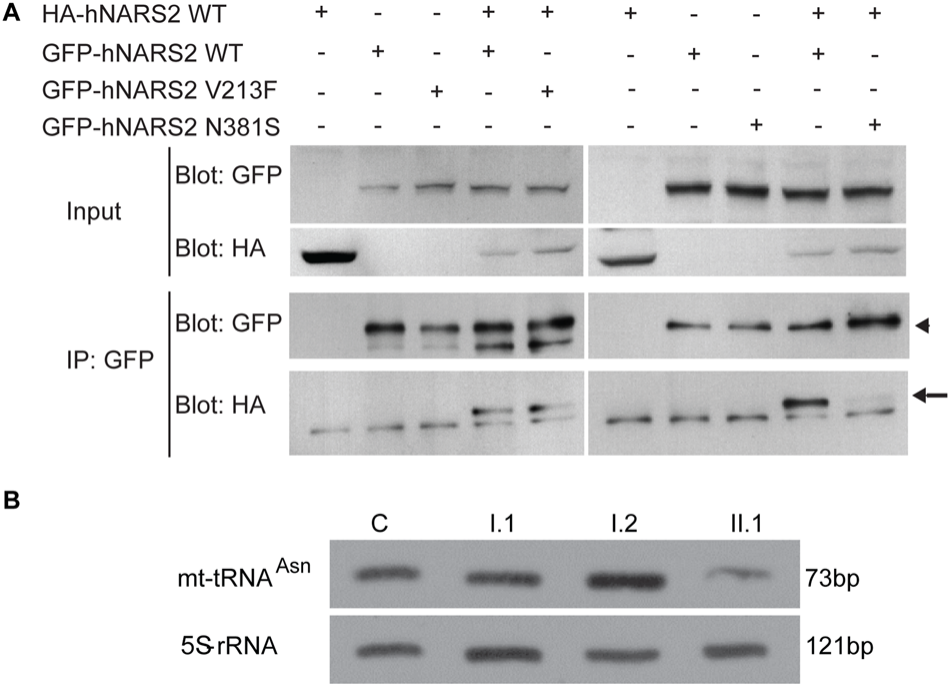
NARS2 homodimerization and RNA level: effect of the p.Val213Phe and p.Asn381Ser mutations. (A) Immunoprecipitates (IP) with anti-GFP antibodies from HEK293T cells transiently transfected with GFP-tagged (arrowhead) and HA-tagged NARS2 (arrow) constructs. Precipitates were immunoblotted with antibodies to the GFP and HA tags. NARS2 homodimerizes, and the p.Val213Phe mutation does not affect the dimerization process. No dimerization was detected with p.Asn381Ser NARS2 construct. (B) Steady state level for mt-tRNA^Asn^ was assessed by Northern blot and the results were validated by two independent laboratories. 5S-rRNA probe was used as a loading control on the same membrane. In fibroblasts of patient II.1, from LS06 family, the level of mt-tRNA^Asn^ is decreased compared to his parents and a control sample. Due to high passage number, we could not measure the mt-tRNA^Asn^ levels in the fibroblast of patient II.3.

Most class II aminoacyl tRNA synthetases function as homodimers [29,36,37]. Extensive studies in autosomal dominant *GARS* related Charcot-Marie-Tooth disease type 2D have shown that tRNA charging is dependent on GARS protein dimerization [36,38]. We therefore wanted to assess whether NARS2, like its cytosolic counterpart would form homodimers. Additionally we wanted to assess whether the missense mutations would disrupt protein conformation sufficiently to alter dimerization with a wild type monomer. For this, we performed co-immunoprecipitation studies using GFP- and HA- tagged wild type and mutant proteins that were co-expressed in HEK293T cells. These studies revealed that *in vitro* wild type NARS2 can homodimerize (Fig. 4D, S5 Fig) and the p.Asn381Ser mutation changes protein structure sufficiently to affect the dimerization with the wild type monomer (Fig. 4D) while the p.Val213 variant does not.

### Neither missense mutation affects *NARS2* expression, stability or mitochondrial targeting in heterologous cells

To determine the effect of the p.Val213Phe and p.Asn381Ser missense mutations on the expression and stability of NARS2, we transiently transfected HEK293T cells with GFP-tagged cDNA constructs encoding either wild type or mutant NARS2. Western Blot analysis and quantification, after normalizing against the GAPDH expression level, revealed no significant difference in the steady state levels of the p.Val213Phe or p.Asn381Ser mutant proteins compared with wild type NARS2 protein (S6D Fig).

To determine if the pathogenic mutations have an effect on the cellular location of NARS2, we performed immunohistochemistry analysis in COS7 cells transiently transfected with GFP-tagged wild type and mutant *NARS2* cDNA constructs (S6A-C Fig). Confocal imaging of the MitoTracker Red FM and GFP-tagged protein revealed an overlap of both signals, which demonstrates the mitochondrial localization of NARS2 (S6A Fig), which is not affected by the p.Val213Phe and p.Asn381Ser alleles (S6B-C Fig). Identical results were obtained with a C-terminal HA tagged wild type and mutant *NARS2* constructs (S7 Fig).

### NARS2 levels are decreased in fibroblasts from family LS06

In order to assess *in vivo* protein stability due to *NARS2* variants in the LS06 family, we performed SDS PAGE and Western blot analysis on whole fibroblast cell lysates from both parents and the probands (Fig. 1C). A truncated NARS2 protein product (Δ154aa) stemming from the p.Tyr323* allele was not observed (Fig. 1C, black arrow). Both probands had significantly reduced NARS2 levels when compared to controls (C1 and C2; Fig. 1C). Since the probands’ full-length NARS2 bands represented translation solely from the paternal allele, Western blot results deemed the p.Asn381Ser *NARS2* variant as unstable at the RNA or protein level. This is further demonstrated by a 50% reduction in NARS2 levels for the paternal sample (Fig. 1C).

### Compound heterozygous mutations (p.Tyr323* and p.Asn381Ser) decrease steady state levels of mt-tRNA^Asn^

We next examined whether the aminoacylation and steady state levels of mt-tRNA^Asn^ were affected in patient fibroblasts. For this we first analyzed the aminoacylation level of tRNAs in total RNA extract isolated from fibroblast cells obtained from probands II.1 and II.3 from family LS06 as well as their mother (I.2) (S8 Fig). NARS2 capacity of aminoacylation was measured by evaluation of the ratio between charged and uncharged mt-tRNA^Asn^. Our results showed aminoacylation activity of NARS2 to be normal in patient fibroblasts (S8 Fig). We also measured steady state mt-tRNA^Asn^ levels for proband II.1, his parents and a control. When normalized against the loading control (5S-rRNA), reduced steady state level for mt-tRNA^Asn^ for patient II.1 was observed (Fig. 4E).

### Defective MRC is associated with mutations in *NARS2*

To test whether the *NARS2* mutations in family LS06 are directly correlated with MRC (Mitochondrial Respiratory Chain) dysfunction, we reasoned that the reintroduction of wild type *NARS2* would correct a hypothetical defect in mtDNA encoded mitochondrial protein translation. Therefore, we overexpressed wild type *NARS2* in patient fibroblasts. We also reasoned if p.Val213Phe is a pathogenic mutation associated with nonsyndromic hearing loss (DFNB94), it would not rescue the MRC defects observed in family LS06. To test these hypotheses, stable *NARS2* over-expression cell lines were generated by lentiviral transduction of patient II.1 fibroblasts with cDNA constructs encoding human wild type or p.Val213Phe mutant *NARS2* cloned into a pLVX-IRES-tdTomato lentiviral plasmid. Transduced cells were sorted by flow cytometry, and tdTomato-positive cells were selected for NARS2 overexpression. The overexpression of NARS2 proteins was confirmed by SDS PAGE and Western blot (S9 Fig).

**Fig 5:**
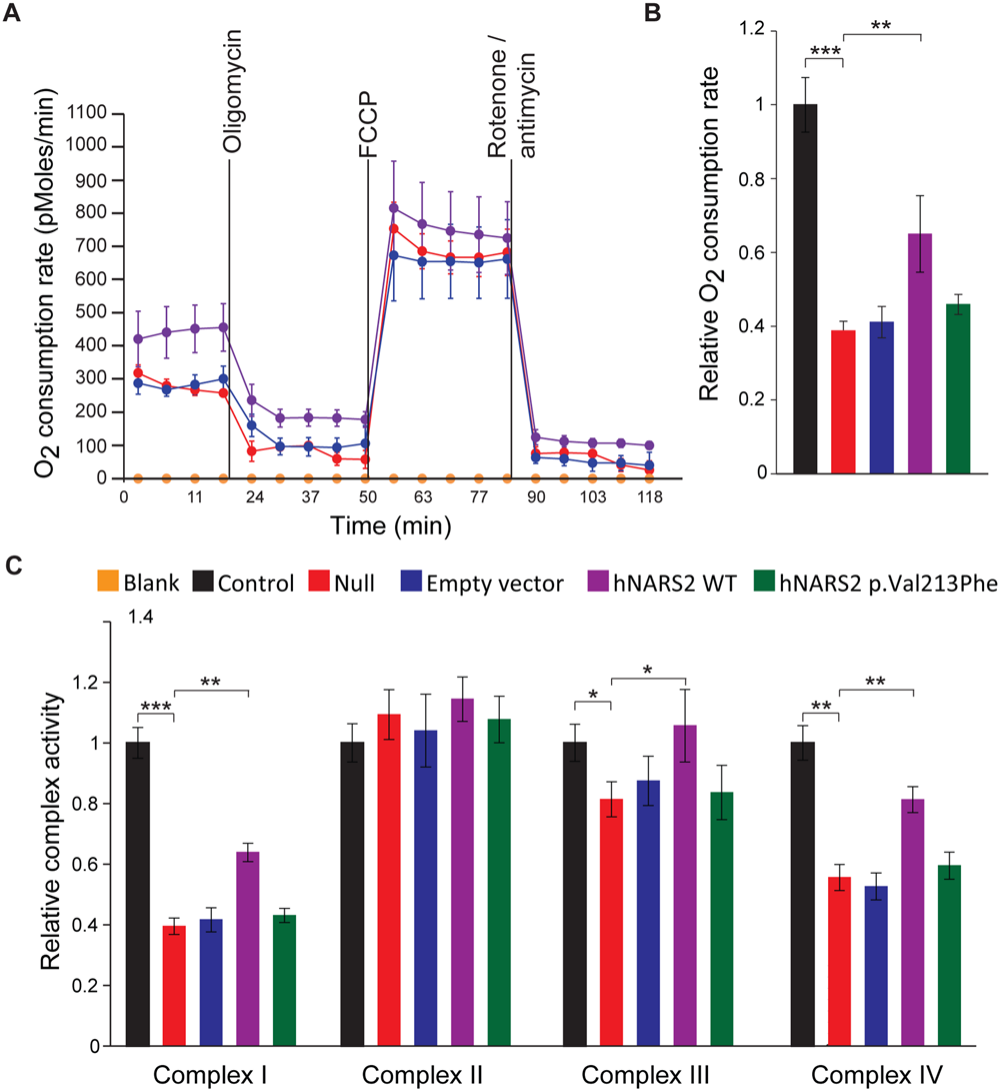
Analysis of the impact of the NARS2 mutations on mitochondrial functions. (A) Oxygen consumption in intact patient cells with NARS2 overexpression. Oxygen consumption in NARS2 overexpressing cells was analyzed using the Seahorse XF24 analyzer. 2 μM oligomycin, 4 μM FCCP and 2 μM rotenone/antimycin were added at the indicated points. (B) Oxygen consumption rates from fibroblast mitochondria of the indicated genotypes. “Null” corresponds to fibroblasts from patient II.1 of LS06 family. Overexpression of the p.Val213Phe NARS2 construct failed to improve the oxygen consumption rate, but the wild type NARS2 construct significantly rescued it (wild type NARS2: OCR ratio = 0.650 ±0.103, p.Val213Phe NARS2: OCR ratio = 0.459 ±0.027, n = 3, p = 0.003). (C) Enzymatic activity of the individual respiratory chain complexes from fibroblast mitochondria of the indicated genotypes. Complex II activity was unaffected in all genotypes. Overexpression of the wild type NARS2 construct significantly improved the activities of complexes I, III and IV (p = 0.002, p = 0.032 and p = 0.004, respectively), and expression of the p.Val213Phe NARS2 construct had no effect. Students t-test have been performed for statistical analysis. Data are represented as the mean ± SEM.

To examine if overexpression of NARS2 rescued the respiratory deficiencies caused by the *NARS2* mutations (p.Tyr323*, p.Asn381Ser), O_2_ consumption of transfectants and their parental cell lines II.1 were measured by using a Seahorse XF24 Analyzer (Fig. 5A-B) and the manufacturer supplied “Mitostress” kit. The kit uses sequential addition of substrates and inhibitors of the mitochondrial respiratory chain. After measuring basal respiration, oligomycin is added to block the mitochondrial ATP synthase. The reduction in oxygen consumption reflects the ATP need of the cell, while the remaining respiration reflects the proton leak. Next the mitochondria are uncoupled with FCCP which causes an increase in electron flux, as the mitochondria attempt to restore the loss of the proton gradient. After addition of FCCP, rotenone and antimycin A are added to inhibit complexes I and III, which then stop all mitochondrial respiration. This allows calculation of respiration due to non mitochondrial oxygen consumption. The oxygen consumption rate (OCR) was significantly higher in the cell line expressing wild type *NARS2* although rescue was not complete (Fig. 5B). These data suggest that the overexpression of *NARS2* can enhance the rate of respiration in the patient cell line carrying the *NARS2* mutations. Furthermore, the transduction with mutant *NARS2* did not rescue the OCR function, which supports the pathogenic nature of the p.Val213Phe allele (Fig. 5B).

Next, we measured the activity of mitochondrial complexes I to IV. The null cell line derived from patient II.1 has decreased activity in complexes I, III and IV. No significant difference was observed in the activity of complex II, which does not have mtDNA encoded subunits. The overexpression of wild type and p.Val213Phe mutant NARS2 did not affect the activity of complex II (Fig. 5C), but the overexpression of wild type NARS2 significantly rescued the activity of complexes I, III and IV in the null fibroblasts (Fig. 5C). Rescue was not complete for mitochondrial complexes I and IV. Contrary to WT construct, the over-expression of the p.Val213Phe mutant NARS2 in null fibroblasts did not rescue the activity of complexes I, II and IV (Fig. 5C). Together with the OCR measurements, the results from these functional studies revealed that Leigh syndrome and DFNB94 associated alleles impair the mitochondrial function of NARS2.

## Discussion

Two brothers diagnosed with Leigh syndrome defined by symmetrical lesions in the brain stem and thalamus, had similar clinical courses including congenital auditory neuropathy followed by myoclonus, intractable seizures and early demise. CSF lactic acid was elevated, suggestive of mitochondrial dysfunction. ETC studies confirmed this suspicion, revealing abnormal complex I/III activity. WES uncovered compound heterozygous mutations in the *NARS2* gene. Compound heterozygous mutations resulted in decreased levels of the tRNA^Asn^, impaired the activities of mitochondrial complexes I, III and IV and negatively affected the oxygen consumption rate in patient fibroblasts. Electron transport through mitochondrial complex II was not enhanced, which is consistent with the fact that the architecture of mitochondrial complex II is completely nuclear encoded. Introduction of wild type NARS2 protein significantly improved OCR and activity of mitochondrial complexes I, III and IV but rescue was incomplete. There could be many reasons for incomplete rescue, including the altered spatiotemporal expression of NARS2, tissue specificity, non-repairable damage to mitochondrial proteins due to endogenous mutant NARS2, cellular health, and genetic modifiers. We have identified a heteroplasmic mt-tRNA^Cys^ variant at position A5793G, located in the tRNA’s acceptor stem. This variant was found once before in a family with epileptic seizures and visual disturbances (personal communication with Dr T. Dorn). However, unaffected maternal relatives in that family also carried the variant. We cannot rule out the possibility that mt-tRNA^Cys^ variant might add to the translational defect and hence resulted in incomplete rescue by overexpression of wild type NARS2.

In the Pakistani family PKDF406, we identified a novel homozygous missense mutation in *NARS2*, which co-segregates with nonsyndromic congenital hearing loss DFNB94. In contrast to wild type NARS2, over-expression of p.Val213Phe NARS2 did not restore OCR or mitochondrial electron transport chain function in fibroblast cells derived from family LS06. Recently, mutations in mt-aaRS (ARS2) and cytoplasmic aaRS (ARS) have been associated with human diseases such as Charcot-Marie-Tooth disease, Perrault syndrome (*LARS2*, *HARS2*) [39,40], and pontocerebellar hypoplasia, all of which include sensorineural hearing impairment (Table 2). The available clinical data indicate that the hearing impairment in the PKDF406 family is nonsyndromic (Table 1). Clinical studies ruled out Charcot-Marie-Tooth disease and pontocerebellar hypoplasia. However, definitively ruling out Perrault syndrome, a disorder of hearing impairment and premature ovarian failure, in affected individuals from family PKDF06 is clinically challenging. Only two females in our family were homozygous for the p.Val213Phe missense mutation and had hearing loss (IV:4 and IV:8; Fig. 1A). At the time of examination, family members IV:4 and IV:8 were 40 and 45 years old, respectively (Table 2), were both post-menopausal, and had no children. The average age for menopause in Pakistan is estimated to be 49.3 years [41], suggesting that these two affected females may have experienced early menopause. The degree of ovarian insufficiency in Perrault syndrome is highly variable and early menopause would be consistent with a diagnosis of Perrault syndrome. We also Sanger-sequenced *NARS2* in 14 unrelated Perrault syndrome probands to assess whether mutations in NARS2 are a common finding in this patient population, but did not find any pathogenic variants.

**Table 2:** Summary of the known aminoacyl tRNA-synthetase genes associated with deafness.

Gene	Localization	Phenotype	Mutations	References	OMIM #
*LARS2*	Mitochondria	Perrault syndrome	p.Thr522Asn p.Ile360Phefs*15 p.Thr629Met	[39]	604544
*HARS2*	Mitochondria	Perrault syndrome	p.Leu200Val p.Val368Leu c.del200–211	[40]	600783
*AARS*	Cytoplasm	Charcot Marie Tooth type 2N	p.Arg329His	[12]	613287
*RARS2*	Mitochondria	Pontocerebellar hypoplasia type 6	p.Met404Lys c.del471–474	[13]	611523
*KARS*	Cytoplasm Mitochondria	Nonsyndromic hearing impairment DFNB89	p.Asp377Asn p.Tyr173His	[14]	613916
*NARS2*	Mitochondria	mild intellectual disability, epilepsy, severe myopathy	p.Gln274His	[8]	612803
		Leigh Syndrome	p.Tyr323*; p.Asn381Ser	This study	
		Nonsyndromic hearing loss	p.Val213Phe	This study	

Pleiotropism is a common phenomenon in ARS associated disease. In our study, comparative mutation and resulting functional deficit analyses for both families may explain the syndromic versus nonsyndromic phenotype. The nonsense Leigh syndrome mutation did not result in a truncated NARS2 protein product in patient fibroblast, which is indicative of nonsense mediated *NARS2* mRNA decay [42]. The remaining NARS2 protein product, expressed from the missense allele, showed additional instability. We were unable to assess stability of p.Val213Phe mutant protein *in vivo* since no patient tissue is available, but the protein was stable *in vitro*. Furthermore the secondary structure of NARS2 harbouring p.Asn381Ser must be significantly affected since the NARS2 p.Asn381Ser protein product has reduced dimerization with wild type NARS2 protein. In contrast, the *DFNB94* allele (p.Val213Phe) did not alter NARS2 protein stability, or the ability to dimerize with wild type protein *in vitro*.

During the course of our work, two siblings with variable clinical manifestation and c.822G>C (p.Gln274His) mutation in *NARS2* were reported by Vanlander and coauthors [8]. One affected individual displays mild intellectual disability and epilepsy, while another sibling has severe myopathy, fatigability and ptosis [8]. Neither sibling has a hearing loss. The.822G<C allele also results in abnormal splicing of intron 7 in patient lymphoblastoid cell lines. In the patient cell lines, NARS2 protein level is somewhat decreased, but is completely absent in muscle from both affected siblings suggesting additional instability of the abnormal splice product in that tissue. To explain the mild phenotype, particularly of the sibling without myopathy, the authors suggest the presence of normally spliced NARS2 protein product below the detection level in muscle and a low threshold for essential NARS2 enzymatic activity. An ETC assay, performed in skeletal muscle homogenate as well as isolated mitochondria shows significantly reduced COI activity. The different methodology makes a direct comparison with our study difficult, but absence of COI/COIII activity in muscle homogenate of the Leigh syndrome patients is striking and suggests a more severe COI defect than what was observed by Vanlander and co-authors. We also observed decreased mt-tRNA^Asn^ levels in our patient fibroblasts, which may have further increased severity of the mitochondrial dysfunction, particularly in CNS tissue. The decreased levels of mt-tRNA^Asn^ may be suggestive either of a regulatory role of NARS2 in the expression of its cognate tRNA or of a decrease in stability of the tRNA by poor interaction with the mutant NARS2 [43]. Fluctuations in mitochondrial tRNA levels have significant influence on disease expression. The best example is reversible infantile respiratory chain deficiency (RIRCD) due to a homoplasmic mutation in the mt-tRNA^Glu^. The mutation may cause lethality in infancy and is associated with low levels of mt-tRNA^Glu^. However, as mt-tRNA^Glu^ levels increase surviving infants spontaneously recover, with good prognosis [44,45]. Decreased mt-tRNAs levels secondary to defects in a mt-tRNA’s cognate *ARS2*, have been observed in several other *ARS2*-related diseases [13,46]. Intriguingly, recent studies have shown that overexpression of cognate and non-cognate ARS2 can rescue mitochondrial dysfunction secondary to mt-tRNA mutations [47,48], further reinforcing the importance of the interaction between ARS2 and their cognate mt-tRNAs.

The absence of hearing deficit in the family studied by Vanlander and co-authors, while the two families described in our study exhibit hearing loss, is an additional example of the pleiotropism of ARS2 associated disease. Interestingly mutations in *KARS* show phenotypic variability to an extent comparable to our findings associated with *NARS2* defects. In three independent studies, different mutations in *KARS* lead to Charcot Marie Tooth disease [12], non syndromic hearing impairment DFNB89 [14] and more recently visual impairment and progressive microencephaly [15]. The link between ARS mutations and phenotypes still need to be unraveled and require a better understanding of the symptoms as well as the involved molecular mechanisms [49].

The phenotypic diversity can also be explained by tissue specificity. Consistent with defects in other mitochondrial disorders [49], and more particularly with other ARS2 disorders (for review [50]), our patients display significant tissue specificity with prominent central nervous system and/or inner ear pathogenicity, despite an ubiquitous expression. Molecular mechanisms underlying this tissue specificity are poorly characterized. Mitochondrial translation might be sufficiently supported by residual ARS2 activity in most of the tissues but may have reached threshold in affected tissue(s) and may depend on the availability of mitochondrial chaperone proteins, in a tissue specific manner [51]. Differential expression of other molecules of the mitochondrial protein synthesis machinery in response to the altered steady state levels of the mutated protein was also shown to be tissue dependent [52]. Significant tissue specific variation of protein stability has previously been reported in fatal mitochondrial hepatopathy due to mutations in the mitochondrial translation elongation factor *GFM1* [52]. While *GFM1* is ubiquitously expressed, the effect of the mutations on GFM1 protein levels was shown to vary from no protein in liver, to 60% of normal protein levels in heart. This finding was consistent with absence of cardiac findings in the patient. Differential expression of other molecules of the mitochondrial protein synthesis machinery in response to the altered steady state levels of GFM1 protein was also tissue specific [52]. The *NARS2* mutations described in this report may therefore affect NARS2 protein stability compensatory gene expression in a tissue specific manner. Several studies have, recently, highlighted the influence of modifying factors and more particularly factors that would modulate the mitochondrial translation [53,54]. Additional function(s) of ARS2 proteins, yet to be described, may be restricted to specific cell types or to specific developmental stages. The fact that in many mt-aaRS related disorders even severe mutations do not display an aminoacylation defect has prompted further studies which have highlighted the importance of the aminoacylation process [5,13,55], protein folding [56,57], and refolding [58]. Therefore the NARS2 mutations described in this study may disrupt protein refolding in the mitochondrial matrix in a tissue-specific manner.

In conclusion, our findings implicate mutant alleles of *NARS2* as another cause of Leigh syndrome as well as DFNB94 hearing loss in humans. Animal models are needed to elucidate the crucial functional roles that NARS2 play in the inner ear and central nervous system as well as in the mitochondrial respiratory chain. Future studies will explore the mechanistic differences in tissue-specific phenotypic expression of *NARS2* mutations causing Leigh syndrome, hearing loss, epilepsy and intellectual disability.

## Materials and Methods

### Linkage analysis

For family PKDF406, screening for linkage to the reported recessive deafness loci was performed using at least three short tandem repeat (STR) markers each for these loci. Data was analyzed using GeneMapper software (Applied Biosystems). LOD scores were calculated using a recessive model of inheritance assuming a fully penetrant disorder and a disease allele frequency of 0.001.

### Whole exome sequencing

For family LS06, genomic DNA was extracted from fibroblast cell lines using the Gentra-Puregen kit (Qiagen) according to the manufacturer’s instructions. Exon capture and enrichment was performed (Illumina TruSeq Exome) in solution and libraries were sequenced as 100 bp paired end reads on an Illumina HiSeq 2000 instrument. Mapping to the human reference sequence hg19 and variant calling was performed using CLC bio genomics workbench 6.5.1 employing the “default stand-alone mapping” algorithm. Variant calling parameters were minimally modified with a neighborhood radius of 9 bp, minimum allele frequency of 35% and minimum coverage of 4 bidirectional reads (Mapping statistics are summarized in S7 Table). Coding region and splice junction variants (6bp) common to probands II.1 and II.3 were filtered using the Annovar “filtered annotation” functions tool [59]. We discarded variants with Minor Allele Frequency (MAF) ≥ 0.01 present in the “1000 genome project April 2012”, “dbSNP138-non flagged” and “ensembl annotations popfreq all” databases. We then selectively analyzed variants compatible with X-linked or autosomal recessive inheritance (S3 Table).

For family PKDF406 whole exome sequencing analysis used a genomic DNA sample from one affected individual (NimbleGen SeqCap EZ Exome Library v2.0, Roche) and 100bp paired-end sequencing was performed on an Illumina HiSeq2000 instrument. Sequencing data were analyzed following the guidelines that are outlined in the Broad Institute’s Genome Analysis Toolkit [60,61]. The raw data were mapped using the Burrows Wheeler Aligner [61], the variants were called using the Unified Genotyper, and the data underwent further processing and quality control [60,61]. Low-quality reads (less than 10x coverage) were removed, and the remaining variants were filtered against the dbSNP133 database and all of the known variants in the NHLBI 6500 Exome Variant database that had a minor allele frequency (MAF) of greater than 0.05% (S3 Table). We used Sanger sequencing to analyze the segregation of alleles in the other family members. Primers were designed with Primer3 software [62] to amplify exons as well as flanking introns and untranslated regions (S4 Table). PCR amplified products were purified with exonuclease and alkaline phosphatase (Fermentas) treatments. Purified products were then sequenced with BigDye v3.1 (Applied Biosystems) and run on an Applied Biosystems 3730xl DNA Analyzer.

### Animals

Post-natal day 0 (P0), P30 and P90 C57BL/6J mice were used. The mice were obtained from Jackson Laboratories and bred in Cincinnati Children’s Hospital Medical Center (CCHMC) animal facility.

### Molecular modeling

Two homology models of NARS2 were constructed, using PYMOL (see Web Resources). The templates, used as backbone, were the crystal structure of *Pyrococcus horikoshii* AsnRS [35] (Protein Data Bank access # 1X55) and the dimeric protein form of mt-AsnRS of *Entamoeba histolytica* (Protein Data Bank access # 3M4Q).

### Nars2 expression analysis

Mouse inner ear tissues were harvested from three to five mice at P0, P30, and P90. The vestibular system and the cochlea were separately dissected and immediately frozen in TriReagent (Ambion). RNA were isolated with RiboPure kit (Ambion) and used to synthetize cDNA (SuperScriptII Reverse Transcriptase, Life Technologies). Inter-exonic primers (S5 Table) were designed with Primer3 software and PCR amplifications were performed with EconoTaq (Lucigen). Primers that amplify *Gapdh* cDNA were used as an internal control (S5 Table). Amplimers were size-separated on a 2% agarose gel and stained with ethidium bromide.

*In situ* hybridization was performed on mouse cochlear cryosections at post-natal day 2 (P2). *Nars2 in situ* hybridization probes were subcloned from C57BL/6J inner ear cDNA and ligated into pCRII-TOPO vector (Invitrogen) for *in vitro* transcription. *Nars2* was detected with a ~1.5 kb probe transcribed from NM_153591, nt501–2078 (S5 Table). *In situ* hybridization positive control probes for *Myo15a* and *NF68* were used as described previously [26,27]. RNA hybridization was performed on 12 μm cryo-sections according to standard methods using digoxygenin-labeled probes in weakly acidic hybridization buffer (pH 4.5), anti-digoxigenin-AP Fab fragments (Roche) in TBST buffer, and the NBT/BCIP colorimetric substrate reaction in AP buffer at pH 9.5.

### Cell culture

HEK293T and COS7 cells were grown in DMEM that was supplemented with 10% FBS, 2 mm glutamine, and penicillin/streptomycin (50 U/ml) (Life Technologies) and were maintained at 37°C in 5% CO_2_. Human Fibroblast cells were grown in DMEM (Gibco) supplemented with 10% fetal bovine serum at 37°C and with 5% CO_2_.

### Generation and analysis of cybrids cell lines

Fibroblast cells were grown in DMEM medium supplemented with glutamax (446 mg/l), 10% fetal calf serum, 50μg/ml uridine and 1mM sodium pyruvate under standard conditions. Cells were fused with 143B rhoo cells as previously described [63]. Several clones were isolated and the resulting cybrid cells were subsequently expanded. Biochemical assays were performed on isolated mitochondria and/or permeabilized cells. Western blot analysis of a patient’s muscle biopsy sample and Blue Native gel analysis (BNG) of a patient’s fibroblast were performed as previously described [63]. We used “MitoProfile” antibody mix (Total OXPHOS human WB antibody cocktail, Abcam) for muscle lysates. For fibroblast lysates we used antibody GRIM19 (Abcam), which corresponds to the mitochondrial complex I subunit NDUFA13.

### Generation of NARS2 stable over-expression cell

The insert of *NARS2* cDNA was cloned into pLVX-IRES-tdTomato lentiviral expression vector using In-Fusion Cloning (Clontech). The construct was packaged into VSV-G pseudotyped viral particles by transfection of HEK293T cells with packaging plasmids and the expression vector for *NARS2*. Lentiviruses were concentrated by ultracentrifugation (CCHMC Vector Core). Transduction of patient fibroblast cell line II.1 was performed according to established methods [64]. Briefly, fibroblast cells from patient were seeded in six-well plates and transduced 22 hours later with 25 μl of concentrated lentivirus (MOI = 6). Polybrene at 4μg/ml was added to increase the transduction efficiency. Transduced cell were sorted by flow cytometry with standard FITC filter sets, and tdTomato positive cells were selected for NARS2 over-expression. Cell sorting was performed at CCHMC Research Flow Cytometry Core with a BD FACSAria II (BD Biosciences).

### Immunofluorescence studies

Wild type (WT) human *NARS2* cDNA (clone #Z7860) was obtained from GeneCopoeia and subcloned into pEGFP-N2 vector (Clontech) using InFusion cloning (Clontech). Stratagene QuikChange Lightning mutagenesis (Roche) was used to introduce the c.637G>T transversion and c.1142A>G transition into WT *NARS2* sequence. All constructs were then sub-cloned in pcDNA3.1(+) (Invitrogen) vector and sequence verified. A HA tag was added at the C-terminal part of NARS cDNA using InFusion cloning.

Constructs were expressed in COS7 cells after transfection with PEI (Polysciences) using a 1:5 ratio (1μg cDNA/5μg PEI). Twenty-four hours post transfection, cells were incubated with 100nM Mito Tracker Red FM (Invitrogen) for 30 minutes, followed by fixation with 4% paraformaldehyde. Fixed cells were mounted with Fluorogel Mounting Medium (EMS) and imaged with a Zeiss LSM700 confocal microscope.

### Immunoprecipitation and western blot

GFP- and HA-tagged NARS2 constructs were co-expressed in HEK293T cells, after transfection using PEI reagent (Polysciences). Forty-eight hours after transfection, cells were harvested and homogenized with sonication in lysis buffer (50mM Tris HCl pH7.4, 100mM NaCl, 1% NP-40, 2mM Na3VO4) containing a protease inhibitor mixture (#P8340, Sigma). Immunoprecipitation was performed with an anti-GFP antibody as described previously [65]. The cell lysates and the immunoprecipitates were processed for Western blot analysis [66]. NARS2 (Abcam), GAPDH (Ambion), GFP (Life Technologies) and HA antibody (Millipore) were used for immunoprecipitation and Western blot analyses.

### Mitochondrial and nuclear DNA analyses

Genomic and mitochondrial DNA was extracted from skeletal muscle, whole blood and primary fibroblasts using Gentra (Quiagen) blood and tissue kits according to the manufacturer guidelines. RNA was extracted from primary fibroblast using Trizol reagent (Life Technologies). The 16 Kb mtDNA genome was PCR amplified in 8 overlapping fragments. Nuclear DNA encoded complex I genes were PCR-amplified from patient cDNA. cDNA was synthesized from RNA from patient fibroblast cells using the iScript cDNA synthesis kit (Biorad). Twenty-nine nuclear complex I subunit genes were amplified. PCR products were purified by ExoSAP-IT (Amersham) and directly sequenced using the PRISMTM Ready Reaction Sequencing Kit (PE Applied Biosystems) on an automatic sequencer (ABI 3130, PE Applied Biosystems). Sequence data were analyzed using Sequencer (version 4.0.5, Genecode Corp.) software.

### Mitochondrial tRNA analysis

Total mitochondrial RNA preparations were obtained from mitochondria isolated from fibroblast cell lines (4.0 × 10^7^ cells). 2μg of total mitochondrial RNA was electrophoresed through a 10% polyacrylamide-7 M urea gel and then electroblotted onto a positively charged nylon membrane (Roche) for hybridization analyses with specific oligodeoxynucleotide probes [67]. Nonradioactive DIG-labeled (Roche) oligodeoxynucleotides (mt-tRNA^Asn^: 5’-CTAGACCAATGGGACTTAAA-3’ and 5S-rRNA: 5’-GGGTGGTATGGCGGTAGAC-3’) were used on the same membrane after stripping in 50% formamide, 5% SDS, 50 mM Tris-HCl, pH 7.5 and extensive washes in 2x SSC [67].

### Mitochondrial tRNA aminoacylation analysis

Total RNA was isolated under acidic conditions. 2μg of total RNA was electrophoresed at 4°C through an acid (pH 5.2) 10% polyacrylamide-7 M urea gel to separate the charged from the uncharged tRNA as detailed elsewhere [68]. The gels were then electroblotted onto a positively charged nylon membrane (Roche) for hybridization analysis with oligodeoxynucleotide probes for mt-tRNA^Asn^ and 5S-rRNA.

### Electron transport chain studies

Muscle and skin samples were obtained during autopsy for the first proband, subject II.1, and from a biopsy at 3 months of age from the second proband, subject II.3, from Caucasian family LS06 with parental consents. Spectrophotometric analysis of the respiratory chain complexes was performed in muscle homogenates through the Center for Inherited Disorders of Energy Metabolism (CIDEM), according to established protocols (Case Western Reserve University, Cleveland, OH).

### Measurements of oxygen consumption rate by Seahorse

Oxygen consumption rate (OCR) measurements were performed by using a Seahorse Bioscience XF-24 instrument (Seahorse Biosciences). Cells were seeded in XF24-well microplates in growth medium and the following day growth medium was replaced with assay medium as described [69].

### Mitochondrial complex I–IV activity assay

Complex I activity was assessed by following the decrease of NADH absorbance at 340 nm, using decylubiquinone as an electron acceptor. The activity of complex II was measured by following the reduction of 2,6-dichlorophenolindophenol (DCPIP) with the decrease of the absorbance at 600 nm of the oxidized DCPIP. Complex III activity was determined by measuring the reduction of cytochrome c by an increase of absorbance at 550 nm. Complex IV activity was measured by monitoring the oxidation of reduced cytochrome c as a decrease of absorbance at 550 nm (Cary 300 UV-Vis Spectrophotometer Agilent, CA.). Complexes I–IV activities were normalized by citrate synthase activity and then used in the analysis [70].

### Study approval

#### Human and animal studies

Approval for this study was obtained from the following institutional review boards (IRBs): University of California Irvine (2002–2608), University of Maryland, School of Medicine, Baltimore, Maryland, USA (HP-00059851), Cincinnati Children’s Hospital Medical Center, Cincinnati, Ohio, USA (2010–0291 and 2013–7868), National Centre of Excellence in Molecular Biology, University of the Punjab, Lahore, Pakistan, and the Combined Neuroscience Institutional Review Board protocol (OH93-DC-0016) at the National Institutes of Health, Bethesda, Maryland, USA. Written informed consent was obtained from all the participating family members.

All experiments and procedures were approved by the Institutional Animal Care and Use Committees of CCHMC and the University of Maryland, School of Medicine.

### Web resources

The URLs for the data presented herein are as follows:

Allen Brain Atlas, http://www.brain-map.org/

ANNOVAR, www.openbioinformatics.org/annovar

DomPred Protein Domain Prediction Server, http://bioinf.cs.ucl.ac.uk/dompred

Online Mendelian Inheritance in Man (OMIM), http://www.omim.org/

NHLBI Exome Sequencing Project (Exome Variant Server), http://evs.gs.washington.edu/EVS/

National Center for Biotechnology Information (NCBI), http://www.ncbi.nlm.nih.gov

Primer3, http://frodo.wi.mit.edu/primer3

PYMOL, www.pymol.org

SHIELD (Shared Harvard Inner-Ear Laboratory Database), https://shield.hms.harvard.edu/

STRAP, http://www.bioinformatics.org/strap/

UCSC Genome Bioinformatics, http://genome.ucsc.edu

Unigene, http://www.ncbi.nlm.nih.gov/unigene/

World Health Organization, http://www.who.int

## Supporting Information

S1 Table Abnormal levels of urine organic acids of patient II.1. (DOCX)

S2 Table Mitochondrial respiratory chain complex activities of muscle homogenate. (DOCX)

S3 Table Summary of exome sequencing analysis for LS06. (DOCX)

S4 Table Summary of exome sequencing analysis for PKDF406. (DOCX)

S5 Table Primer sequences used to amplify mouse *Nars2*. (DOCX)

S6 Table Predicted effect of p.Asn381Ser and p.Val213Phe missense mutations on NARS2. (DOCX)

S7 Table Summary of sequencing statistic for LS06. (DOCX)

S8 Table Primer sequences used to amplify and sequence human *NARS2* coding exons. (DOCX)

S1 Fig Western blot and Blue Native Gel analyses for patient fibroblasts and transmitochondrial cybrids. (A) SDS PAGE followed by Western Blot of fibroblast cell lysate from patient II.1 does not show lower levels of mitochondrial complex I (COI) subunit NDUFA13 (Grim19). Porin is used as a loading control. (B-C) We generated mitochondrial cybrid cell lines for II.1 and I.2 to delineate mtDNA vs nDNA origins of the complex I defect. For this we fused enucleated patient fibroblasts with a human osteosarcoma (143b) rho^0^ cell line and selected clones displaying 5, 10, 95 and 98% of heteroplasmy respectively for mt-tRNA^Cys^ A5793G. Western Blot (B) and BNG analyses (C) for COI (NDUFA13-GRIM19) were both normal for II.1 and I.2 irrespective of heteroplasmy levels.
(TIF)

S2 Fig Audiograms of individuals V.4 and V.8 from family PKDF406. Hearing loss in the affected family members was evaluated by pure-tone audiometry, which tested frequencies that ranged from 250 Hz to 8 kHz. It was determined to be severe to profound, sensorineural and bilateral. The symbols ‘o’ and ‘x’ denote air conduction pure-tone thresholds in the right and the left ears, respectively.
(TIF)

S3 Fig Segregation and Sanger sequencing of mt-tRNACys A5793G. (A) Extended pedigree. (B) A novel mt-tRNACys variant at position A5793G had been shown in the proband and other maternal relatives via full mtDNA sequencing. The variant displays heteroplasmy (varying levels of variant vs wild-type mtDNA). (C) Position of A5793G mt-tRNACys at the base of the acceptor stem. The variant is completely conserved in mammals.
(TIF)

S4 Fig Sequencing chromatograms. (A) Nucleotide sequence chromatograms of exon 6 of *NARS2* comparing the wild type sequence, heterozygosity and homozygosity of the c.637G>T mutation. (B) Nucleotide sequence chromatograms of exons 10 and 11 of *NARS2* comparing the wild type sequence, heterozygosity and coumpond heterozygosity of the c.969T>A and c.1142A>G mutations.
(TIF)

S5 Fig Interaction between HA- and GFP-tags. Immunoprecipitates (IP) with anti-GFP antibodies from HEK293 cells transiently transfected with GFP and HA-tagged NARS2 constructs. Precipitates were immunoblotted with antibodies to the GFP and HA tags. No dimerization was detected between GFP and HA-NARS2 constructs (black arrow).
(TIF)

S6 Fig Impact of the p.Val213Phe and p.Asn381Ser mutations on NARS2 localization and expression level *in vitro*. (A) Wild type NARS2-GFP (green) was transiently expressed in COS-7 cells, and Mito Tracker Red FM was used to stain mitochondria. Co-localization of the fluorescent signals indicates the mitochondrial targeting of wild type NARS2. (B) p.Val213Phe NARS2-GFP (green) and (C) p.Asn381Ser NARS2-GFP (green) were also targeted to mitochondria, indicating that these disease-causing mutations do not affect NARS2 localization. The scale bar is 5 μm and applies to all panels. (D) Immunoblot analysis of transfected GFP-tagged *NARS2* constructs. HEK293 cells were transiently transfected with the same quantity of wild type or mutant *NARS2* constructs. Protein extracts from the cell lysates were analyzed by Western blot using an anti-GFP antibody. The expected size of both GFP-fused proteins is 81 kDa. Wild type, p.Val213Phe and p.Asn381Ser mutant NARS2 appear to be equally expressed in the transfected cells. A GAPDH antibody was used as a loading control.
(TIF)

S7 Fig Effect of the p.Val213Phe and p.Asn381Ser mutations on NARS2 localization. (A-C) The localization of HA-tagged wild type and mutant NARS2 in COS-7 cells. (A) Wild type, (B) p.Val213Phe NARS2-HA construct and (C) p.Asn381Ser NARS2-HA construct were transiently expressed in COS-7 cells. Mito Tracker Red FM was used to stain mitochondria. NARS2 was labeled using a monoclonal HA antibody (green). The two signals co-localized for wild type and mutant NARS2, suggesting that both mutations do not affect NARS2 targeting to the mitochondria. The scale bar is 5 μm and applies to all panels.
(TIF)

S8 Fig Aminoacylation assays for mitochondrial tRNA^Asn^. The aminoacylated tRNAs were separated from nonaminoacylated tRNA species on acidic denaturing polyacrylamide-urea gels and then electro-blotted and hybridized with specific probes for mt-tRNA^Asn^, mt-tRNA^Lys^ and mt-tRNA^Asp^. Samples of mitochondrial tRNA were deacylated by being treated at pH 9. The blot shows normal aminoacylation for mt-tRNA^Asn^ in II.1. Northern Blotting for mt-tRNA^Asn^ levels was performed 3x for RNA from I.2. II.1 and II.3. The data consistently showed normal aminoacylation for both patients while mt-tRNA^Asn,Lys,Asp^ levels varied between experiments and patients and a clear determination would not be made.
(TIF)

S9 Fig SDS-PAGE and western blot analysis of lentiviral transduction and expression of NARS2. *NARS2* lentiviral constructs were made by cloning human *NARS2* cDNA into pLVX-IRES-tdTomato vector and then packaged into pseudoviral particles. NARS2 expression was assessed with Western Blot in transduced patient cells to monitor the transduction efficiency, using an anti-NARS2 antibody. GAPDH antibody was used as a loading control.
(TIF)
