# Supplementary material for: Mutations of Human NARS2, Encoding the Mitochondrial Asparaginyl-tRNA Synthetase, Cause Nonsyndromic Deafness and Leigh Syndrome
Source: PLoS Genet. 2015 Mar 25;11(3):e1005097. doi: 10.1371/journal.pgen.1005097 (PMC4373692; doi:10.1371/journal.pgen.1005097)
Supplement: S1 Table — (DOCX) [file pgen.1005097.s001.docx]

**Table S1: Abnormal levels of urine organic acids of patient II.1.**

| Organic acids | Patient II.1  (mM/mol creatinine) | Reference Ranges  (mM/mol creatinine) |
| --- | --- | --- |
| Benzoic acid | 44 | 0-6 |
| Ethylmalonic acid | 14 | 0-11 |
| Malic acid | 52 | 0-13 |
| Pyruvic acid | 27 | 0-22 |
| 2-OH-glutaric acid | 29 | 0-22 |
| 3-OH-adipic acid | 35 | 0-20 |
| Suberic acid | 9 | 0-7 |
| Citric acid | >1344 | 120-675 |
| Hippuric acid | >3166 | 0-784 |
| OH-decanedioic acid | 7 | 0-2 |
| N-acetyltyrosine acid | 5 | 0-2 |
