# Supplementary material for: Mutations of Human NARS2, Encoding the Mitochondrial Asparaginyl-tRNA Synthetase, Cause Nonsyndromic Deafness and Leigh Syndrome
Source: PLoS Genet. 2015 Mar 25;11(3):e1005097. doi: 10.1371/journal.pgen.1005097 (PMC4373692; doi:10.1371/journal.pgen.1005097)
Supplement: S2 Table — (DOCX) [file pgen.1005097.s002.docx]

**Table S2: Mitochondrial respiratory chain complex activities of muscle homogenate**

| **Complex** | **Enzyme analyzed** | **II.1** | **II.2** |
| --- | --- | --- | --- |
| **I** | NADH-Ferricyanide reductase | 94% | 84% |
| **I / III** | NADH-cytochrome c reductase (rot. sens) | 0.01% | 12% |
| **II** | Succinate dehydrogenase | 112% | 102% |
| **II / III** | Succinate-cytochrome c reductase (antimycin sensitive) | 42% | 71% |
| **III** | Decylubiquinol-cytochrome c reductase | 59% | 68% |
| **IV** | Cytochrome c oxidase | 44% | 103% |
