# Supplementary material for: Mutations of Human NARS2, Encoding the Mitochondrial Asparaginyl-tRNA Synthetase, Cause Nonsyndromic Deafness and Leigh Syndrome
Source: PLoS Genet. 2015 Mar 25;11(3):e1005097. doi: 10.1371/journal.pgen.1005097 (PMC4373692; doi:10.1371/journal.pgen.1005097)
Supplement: S3 Table — (DOCX) [file pgen.1005097.s003.docx]

**Table S3: Summary of Exome sequencing analysis for LS06**

|  | **Exome** | **Gene** |
| --- | --- | --- |
| Variants in common for both probands and with same zygosity  Coding region and splice variants  1000g2012apr_all MAF <0.01 filtering  dbSNP138_NonFlagged threshold 0.01  Ensemble annotations popfreq_all threshold 0.01  X-linked genes  Genes with two or more variants in trans  Genes with mitochondrial association  Mitochondrial clinical variant | 65,339  12,168  1,456  181  90  3  2  1  1 | *Gria3, IRS4, MAGEC1*  *ANKRD36, NARS2*  *NARS2*  *Mfn2* |
