# Supplementary material for: Mutations of Human NARS2, Encoding the Mitochondrial Asparaginyl-tRNA Synthetase, Cause Nonsyndromic Deafness and Leigh Syndrome
Source: PLoS Genet. 2015 Mar 25;11(3):e1005097. doi: 10.1371/journal.pgen.1005097 (PMC4373692; doi:10.1371/journal.pgen.1005097)
Supplement: S4 Table — (DOCX) [file pgen.1005097.s004.docx]

**Table S4: Summary of Exome sequencing analysis for PKDF406**

|  | **Exome** |
| --- | --- |
| Total changes  Changes not in dbSNP135 (MAF < 1%)  Non-synonymous/Splice site/insertions/deletions  Homozygous/compound heterozygous changes  Changes not found in Pakistani control samples  Changes not found in 1000 genome or NHLBI ESP  Potential pathogenic changes in known deafness genes  Changes present in the *DFNB94* linkage interval  Changes predicted to be pathogenic^#^  Changes segregating with hearing loss in family PKDF468 | 61,250  2359  352  31  21  21  0  1  1  1 |

^#^Pathogenic predicted by at least two of the four prediction programs: Polyphen-2, SNPs3D, MutationTaster and SIFT.
