# Supplementary material for: Mutations of Human NARS2, Encoding the Mitochondrial Asparaginyl-tRNA Synthetase, Cause Nonsyndromic Deafness and Leigh Syndrome
Source: PLoS Genet. 2015 Mar 25;11(3):e1005097. doi: 10.1371/journal.pgen.1005097 (PMC4373692; doi:10.1371/journal.pgen.1005097)
Supplement: S5 Table — (DOCX) [file pgen.1005097.s005.docx]

**Table S5: Primer sequences used to amplify mouse *Nars2***

|  | Forward Primer | Reverse Primer | Product (bp) |
| --- | --- | --- | --- |
| *mNars2* | TGCACATTCATACCCCAGTAC | ACCATCTTCATTCTCCCTCATG | 626 |
| *mGAPDH* | TCAACAGCAACTCCCACTCTTCCA | ACCCTGTTGCTGTAGCCGTATTCA | 115 |
| Riboprobe | TTGTTGCGGATTCAAGCTTCG | CCCTGAGGAACGCTGAGA | 1579 |
