# Supplementary material for: Mutations of Human NARS2, Encoding the Mitochondrial Asparaginyl-tRNA Synthetase, Cause Nonsyndromic Deafness and Leigh Syndrome
Source: PLoS Genet. 2015 Mar 25;11(3):e1005097. doi: 10.1371/journal.pgen.1005097 (PMC4373692; doi:10.1371/journal.pgen.1005097)
Supplement: S6 Table — (DOCX) [file pgen.1005097.s006.docx]

**Table S6: Predicted effect of p.Asn381Ser and p.Val213Phe missense mutations on NARS2**

| ***In silico* pathogenicity**  **prediction tool** | **p.Asn381Ser predicted**  **effect (score)** | **p.Val213Phe predicted**  **effect (score)** |
| --- | --- | --- |
| ^1^PolyPhen-2 HDIV | **Prob. damaging (0.999)** | **Prob. damaging (0.978)** |
| ^2^PolyPhen-2 HVAR | **Prob. damaging (0.978)** | Poss. damaging (0.815) |
| ^3^LRT | **Deleterious (1)** | **Deleterious (1)** |
| ^4^Mutation Taster | **Disease causing (1)** | **Disease causing (1)** |
| ^5^SIFT | **Deleterious (0.02)** | **Deleterious (0)** |
| ^6^Mutation Assessor | **Medium 1.98** | **Medium (2.045)** |
| ^7^Provean | **Deleterious (-4.42)** | Neutral (-2.3) |
| ^8^FATHMM | **Damaging (-1.82)** | Tolerated (-1.27) |
| ^9^RadialSVM | **Deleterious (0.497)** | Tolerated (-0.075) |
| ^10^LR | **Deleterious (0.696)** | Tolerated (0.482) |
| ^11^GERP++ | 4.59 | 3.89 |

**^1^**Scores range from 0-0.452 (Benign) 0.453-0.956 (possibly damaging) 0.957 -1 (probably damaging)

**^2^**Scores range from 0-0.446 (Benign) 0.447-0.909 (possibly damaging) 0.909 -1 (probably damaging)

**^3,4,5^**Scores range from 0-1;

^6^Scores range from neutral, low, medium to high

^7^Scores < -2.5=damaging

^8^Scores < -1.5=damaging

^9,10^Scores > 0.5=damaging

^11^Scores increase with degree of conservation
