# Supplementary material for: Mutations of Human NARS2, Encoding the Mitochondrial Asparaginyl-tRNA Synthetase, Cause Nonsyndromic Deafness and Leigh Syndrome
Source: PLoS Genet. 2015 Mar 25;11(3):e1005097. doi: 10.1371/journal.pgen.1005097 (PMC4373692; doi:10.1371/journal.pgen.1005097)
Supplement: S7 Table — (DOCX) [file pgen.1005097.s007.docx]

**Table S7: Summary of sequencing statistic for LS06**

| Sample | Total reads | Mapped reads | % mapped reads | % reads in pair | Average read length | Average length for paired reads | Cov 1x (%) | Cov 5x (%) | Cov 10x (%) | Cov 20x (%) |
| --- | --- | --- | --- | --- | --- | --- | --- | --- | --- | --- |
| I.1 | 246,260,344 | 242,668,234 | 98.5 | 95.9 | 105 | 221 | 96.3 | 94.6 | 93.1 | 90.6 |
| II.1 | 192,208,754 | 163,146,325 | 84.9 | 71.9 | 105 | 262 | 96.4 | 93.8 | 91.6 | 87.7 |
| II.3 | 247,022,204 | 243,329,645 | 98.5 | 95.6 | 105 | 230 | 96.8 | 94.6 | 93 | 90.4 |
