# Supplementary material for: Mutations of Human NARS2, Encoding the Mitochondrial Asparaginyl-tRNA Synthetase, Cause Nonsyndromic Deafness and Leigh Syndrome
Source: PLoS Genet. 2015 Mar 25;11(3):e1005097. doi: 10.1371/journal.pgen.1005097 (PMC4373692; doi:10.1371/journal.pgen.1005097)
Supplement: S8 Table — (DOCX) [file pgen.1005097.s008.docx]

**Table S8: Primer sequences used to amplify and sequence human *NARS2* coding exons**

| Exon | Forward Primer | Reverse Primer | Product (bp)^a^ |
| --- | --- | --- | --- |
| 1 | CTCTGGAGTGCCTTAGAGCAGAG | CGACGCCTACACTTTCTAACTTTTC | 399 |
| 2 | GAAGTTGATTTGTTGAGGCAGGAT | TATTCGGAGTTGATCCTCAAAGCTA | 379 |
| 3 | TCTATGGGAGAACTTGAACTCGAAC | TTAACTGTGACACACACCACAGAGA | 820 |
| 4 | CGTCCTACCATTCCACTTACCTG | GCACCCAAAGCTAGGTCATGTAG | 495 |
| 5 | AAAATTATTTGTGCCCCTGTGG | TGTCACAGCATAAGACTGCTGGT | 468 |
| 6 | CCTTTATTTTCCTGAGAACAACCTG | CATGTTTGGGATTCAAGCAAGA | 477 |
| 7 | AGCAAATATGTGTCAGTCTGTGGAC | AATGGCAGAGCTGGGATATAAACTT | 475 |
| 8 | GAATGCGGTCCTTATTGAGTCCT | CCTGGAATCTGGGGAGTGTCTAT | 495 |
| 9 | TGGTTCTCTCAAAATGTCCTGAAG | CCTGGAATCTGGGGAGTGTCTAT | 375 |
| 10 | GTCTTCAGGCAGATCATTCTTTCAT | AAACGTGGGAGACTCCTTTATAACC | 495 |
| 11 | CCTTAGCAGCTAGCTCCACACAT | GACCTTGTGACAGAAATGAGGAAGT | 472 |
| 12 | ACTACACAAATGGTGCAAGAGAGAG | TTGCTCAAGGACATAGAGAATCTGA | 419 |
| 13 | AGAGGCTTTGTCGTCTACTTCTTCA | CCCTTCCCTGACCTAAGAACAGTTA | 286 |
| 14 | TTCTTCCTTTCCATTGTCTGTCTGT | CATCCTTACGTGAAATACCCTCAAC | 500 |

^a^All PCR products were amplified with 1.5 mM MgCl_2_ and 60^o^C annealing temperature.
