# Supplementary figures and images for: Mutations of Human NARS2, Encoding the Mitochondrial Asparaginyl-tRNA Synthetase, Cause Nonsyndromic Deafness and Leigh Syndrome
Source: PLoS Genet. 2015 Mar 25;11(3):e1005097. doi: 10.1371/journal.pgen.1005097 (PMC4373692; doi:10.1371/journal.pgen.1005097)

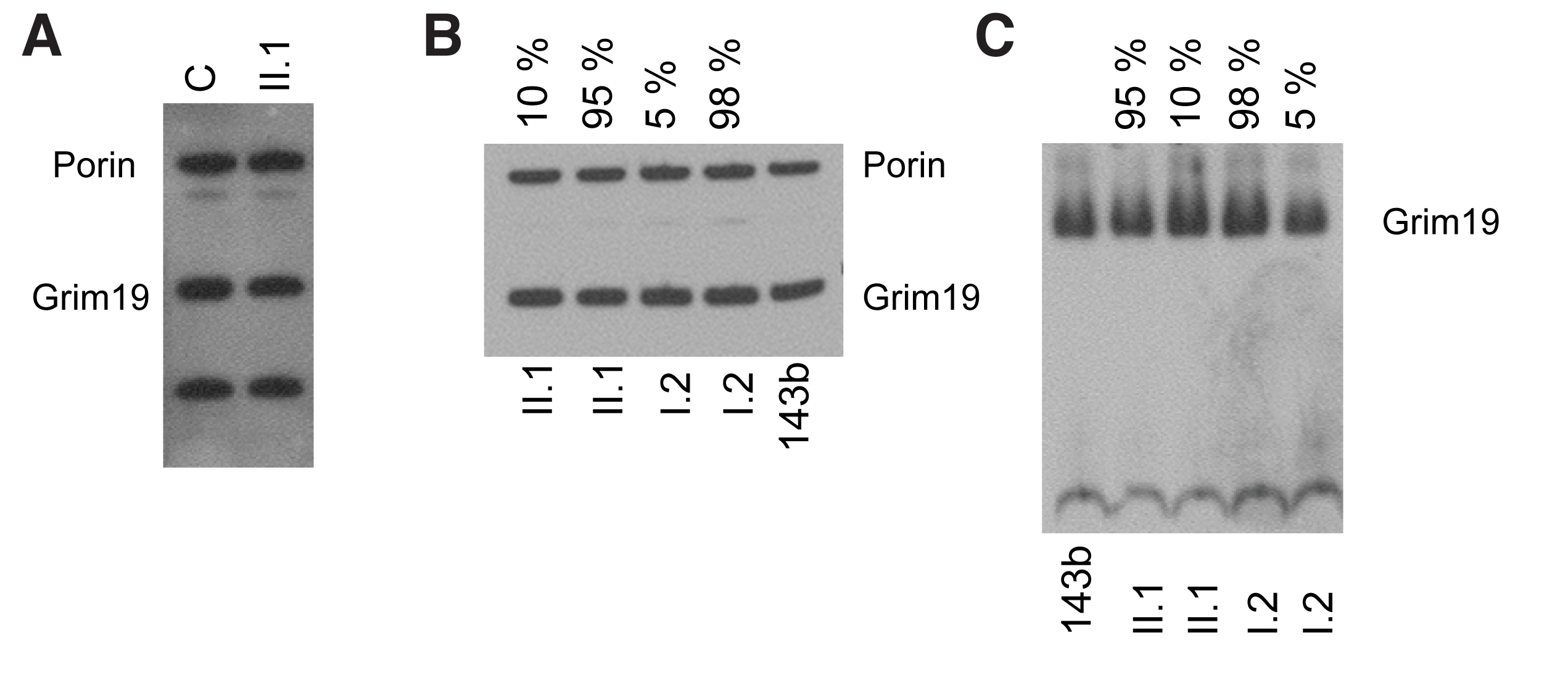

Supplement: S1 Fig — (A) SDS PAGE followed by Western Blot of fibroblast cell lysate from patient II.1 does not show lower levels of mitochondrial complex I (COI) subunit NDUFA13 (Grim19). Porin is used as a loading control. (B-C) We generated mitochondrial cybrid cell lines for II.1 and I.2 to delineate mtDNA vs nDNA origins of the complex I defect. For this we fused enucleated patient fibroblasts with a human osteosarcoma (143b) rho0 cell line and selected clones displaying 5, 10, 95 and 98% of heteroplasmy respectively for mt-tRNACys A5793G. Western Blot (B) and BNG analyses (C) for COI (NDUFA13-GRIM19) were both normal for II.1 and I.2 irrespective of heteroplasmy levels. (TIF) [file pgen.1005097.s009.tif]

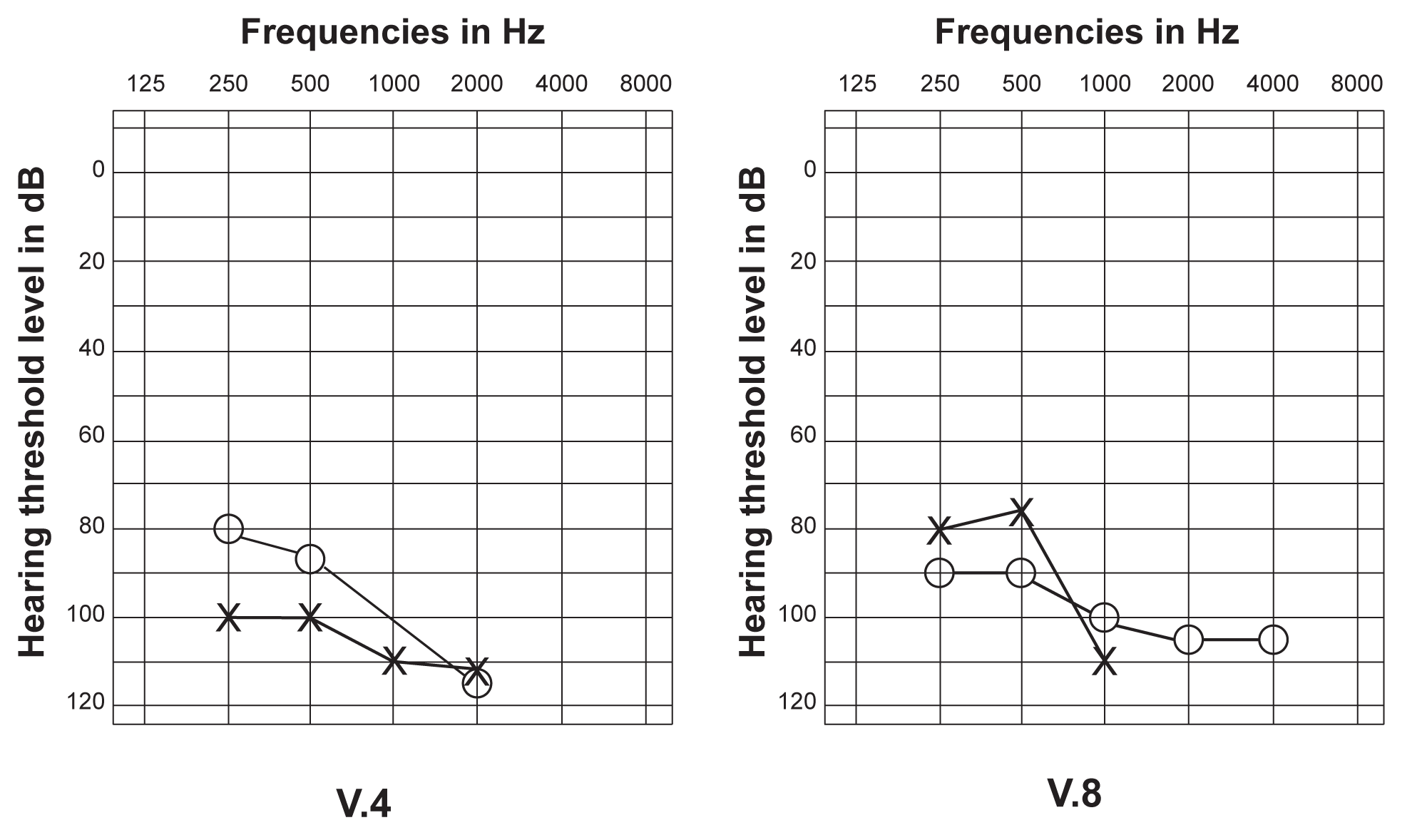

Supplement: S2 Fig — Hearing loss in the affected family members was evaluated by pure-tone audiometry, which tested frequencies that ranged from 250 Hz to 8 kHz. It was determined to be severe to profound, sensorineural and bilateral. The symbols ‘o’ and ‘x’ denote air conduction pure-tone thresholds in the right and the left ears, respectively. (TIF) [file pgen.1005097.s010.tif]

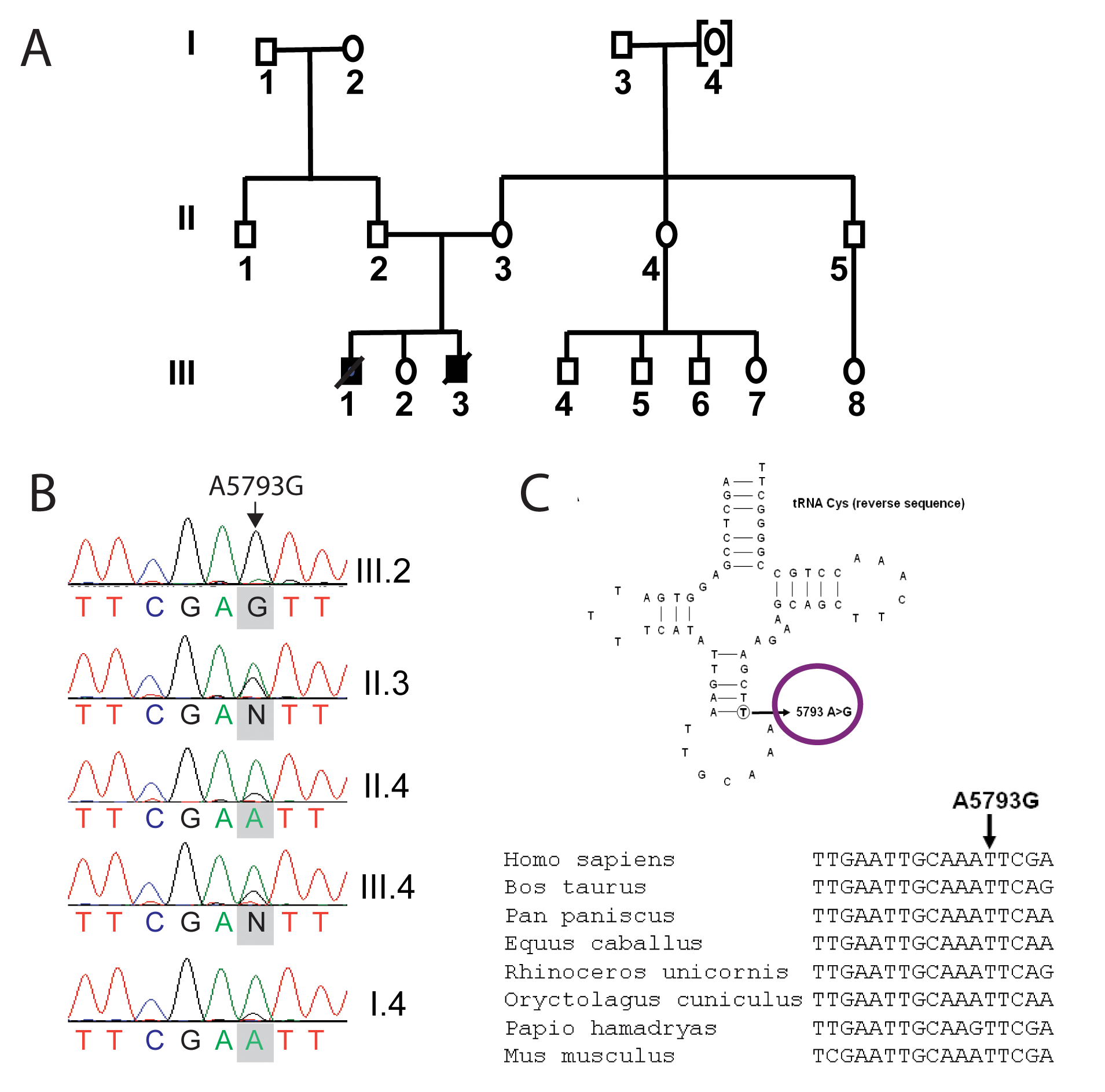

Supplement: S3 Fig — (A) Extended pedigree. (B) A novel mt-tRNACys variant at position A5793G had been shown in the proband and other maternal relatives via full mtDNA sequencing. The variant displays heteroplasmy (varying levels of variant vs wild-type mtDNA). (C) Position of A5793G mt-tRNACys at the base of the acceptor stem. The variant is completely conserved in mammals. (TIF) [file pgen.1005097.s011.tif]

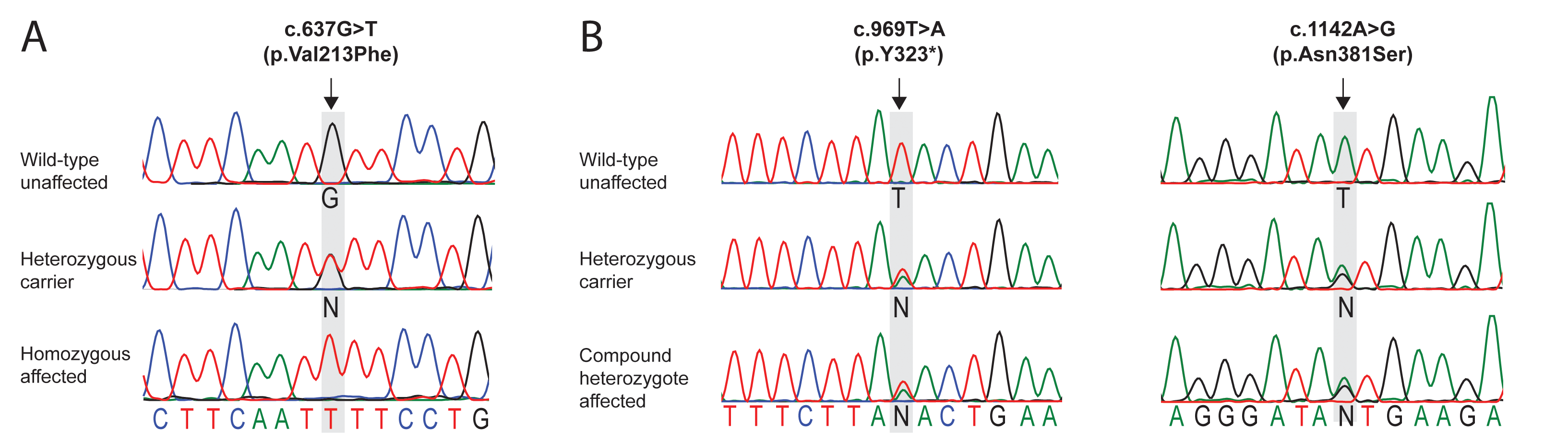

Supplement: S4 Fig — (A) Nucleotide sequence chromatograms of exon 6 of NARS2 comparing the wild type sequence, heterozygosity and homozygosity of the c.637G>T mutation. (B) Nucleotide sequence chromatograms of exons 10 and 11 of NARS2 comparing the wild type sequence, heterozygosity and coumpond heterozygosity of the c.969T>A and c.1142A>G mutations. (TIF) [file pgen.1005097.s012.tif]

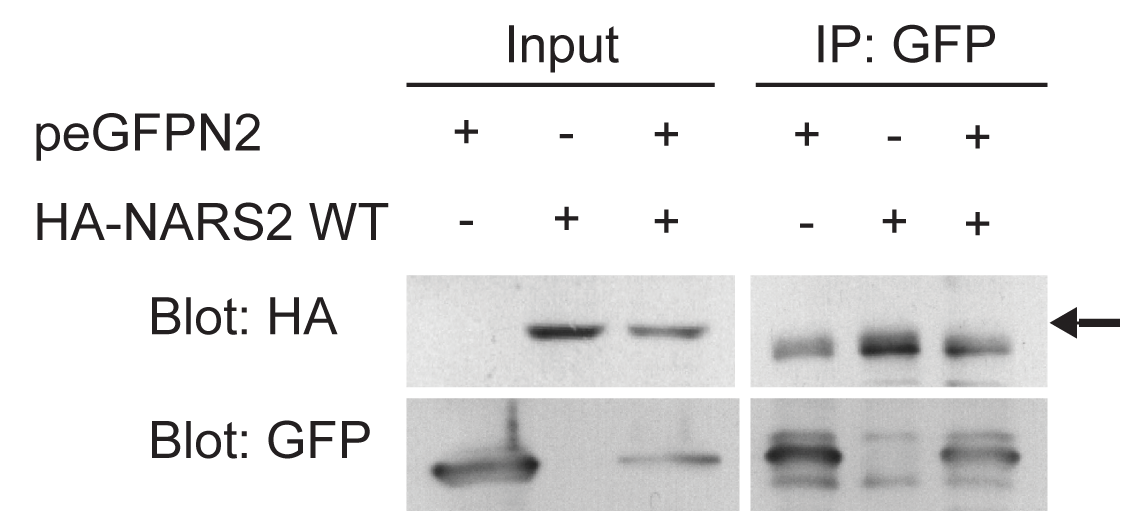

Supplement: S5 Fig — Immunoprecipitates (IP) with anti-GFP antibodies from HEK293 cells transiently transfected with GFP and HA-tagged NARS2 constructs. Precipitates were immunoblotted with antibodies to the GFP and HA tags. No dimerization was detected between GFP and HA-NARS2 constructs (black arrow). (TIF) [file pgen.1005097.s013.tif]

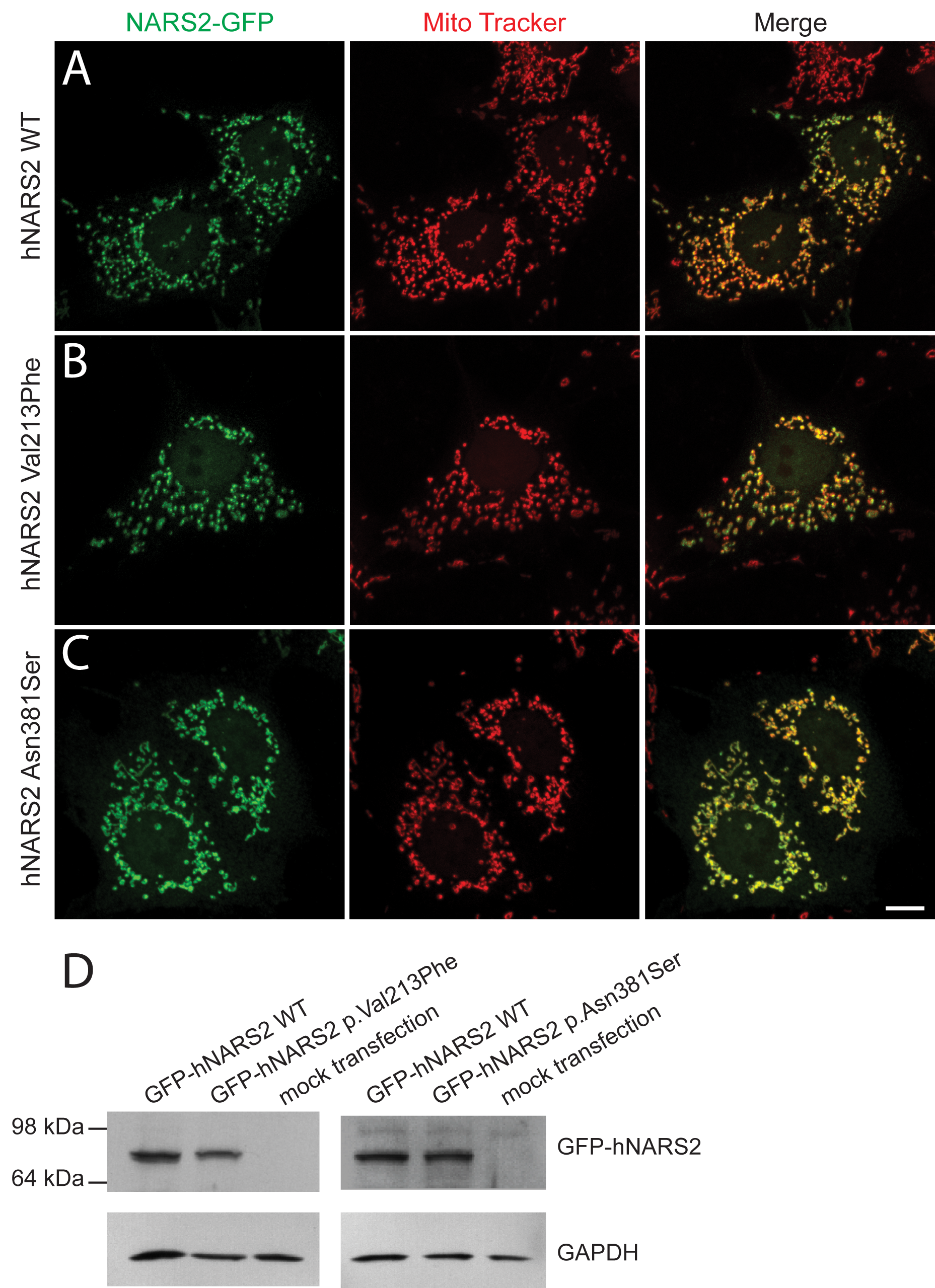

Supplement: S6 Fig — (A) Wild type NARS2-GFP (green) was transiently expressed in COS-7 cells, and Mito Tracker Red FM was used to stain mitochondria. Co-localization of the fluorescent signals indicates the mitochondrial targeting of wild type NARS2. (B) p.Val213Phe NARS2-GFP (green) and (C) p.Asn381Ser NARS2-GFP (green) were also targeted to mitochondria, indicating that these disease-causing mutations do not affect NARS2 localization. The scale bar is 5 μm and applies to all panels. (D) Immunoblot analysis of transfected GFP-tagged NARS2 constructs. HEK293 cells were transiently transfected with the same quantity of wild type or mutant NARS2 constructs. Protein extracts from the cell lysates were analyzed by Western blot using an anti-GFP antibody. The expected size of both GFP-fused proteins is 81 kDa. Wild type, p.Val213Phe and p.Asn381Ser mutant NARS2 appear to be equally expressed in the transfected cells. A GAPDH antibody was used as a loading control. (TIF) [file pgen.1005097.s014.tif]

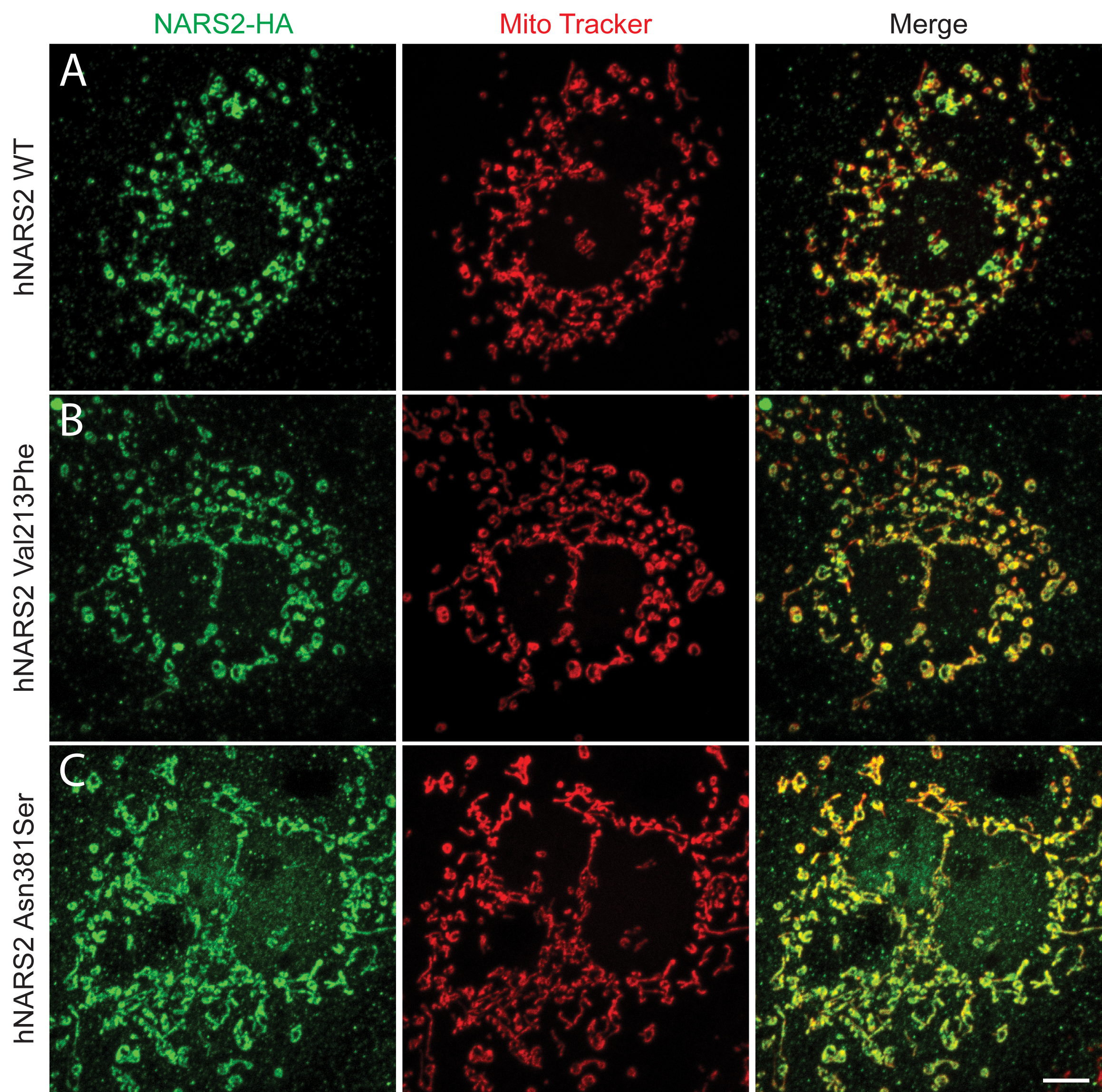

Supplement: S7 Fig — (A-C) The localization of HA-tagged wild type and mutant NARS2 in COS-7 cells. (A) Wild type, (B) p.Val213Phe NARS2-HA construct and (C) p.Asn381Ser NARS2-HA construct were transiently expressed in COS-7 cells. Mito Tracker Red FM was used to stain mitochondria. NARS2 was labeled using a monoclonal HA antibody (green). The two signals co-localized for wild type and mutant NARS2, suggesting that both mutations do not affect NARS2 targeting to the mitochondria. The scale bar is 5 μm and applies to all panels. (TIF) [file pgen.1005097.s015.tif]

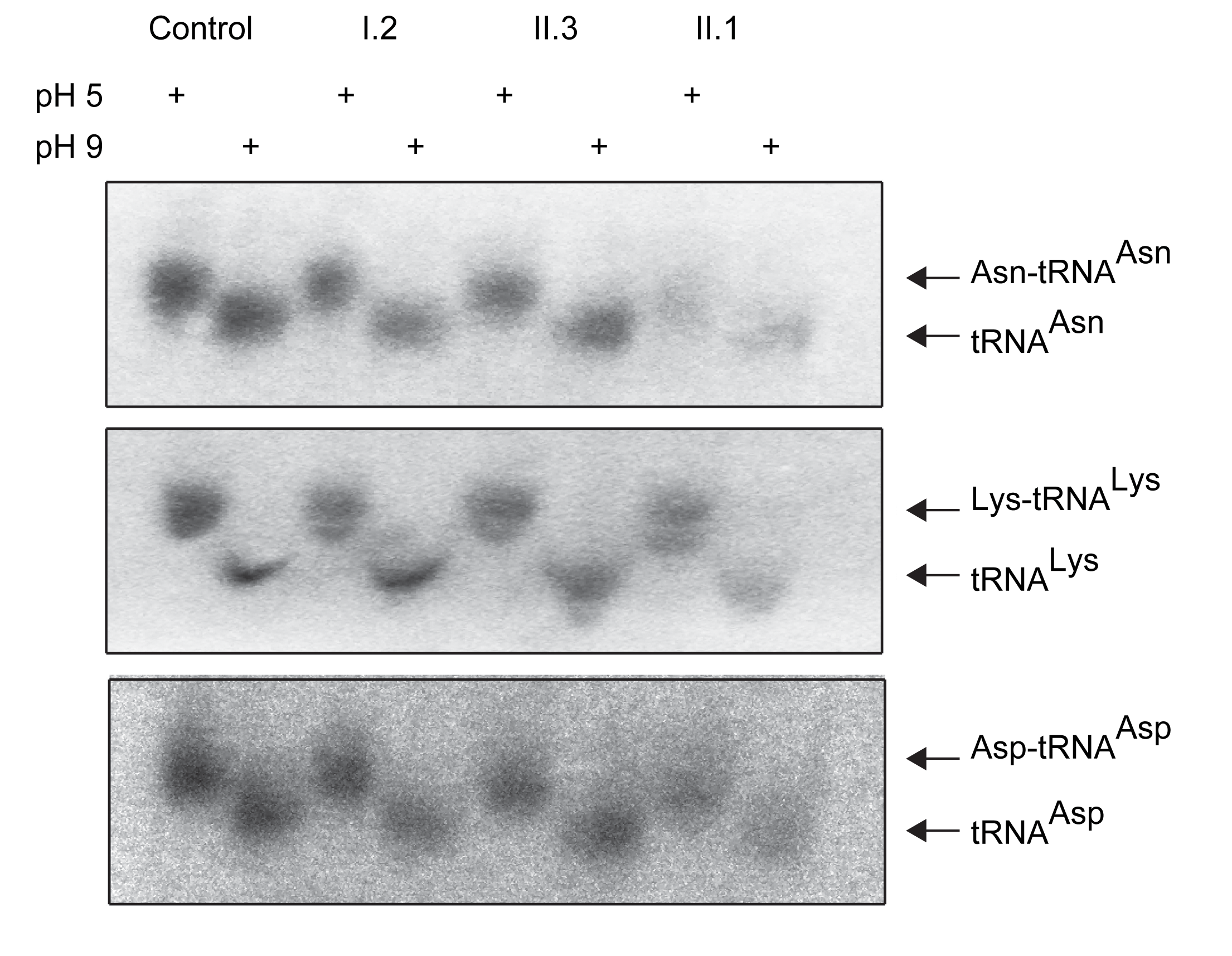

Supplement: S8 Fig — The aminoacylated tRNAs were separated from nonaminoacylated tRNA species on acidic denaturing polyacrylamide-urea gels and then electro-blotted and hybridized with specific probes for mt-tRNAAsn, mt-tRNALys and mt-tRNAAsp. Samples of mitochondrial tRNA were deacylated by being treated at pH 9. The blot shows normal aminoacylation for mt-tRNAAsn in II.1. Northern Blotting for mt-tRNAAsn levels was performed 3x for RNA from I.2. II.1 and II.3. The data consistently showed normal aminoacylation for both patients while mt-tRNAAsn,Lys,Asp levels varied between experiments and patients and a clear determination would not be made. (TIF) [file pgen.1005097.s016.tif]

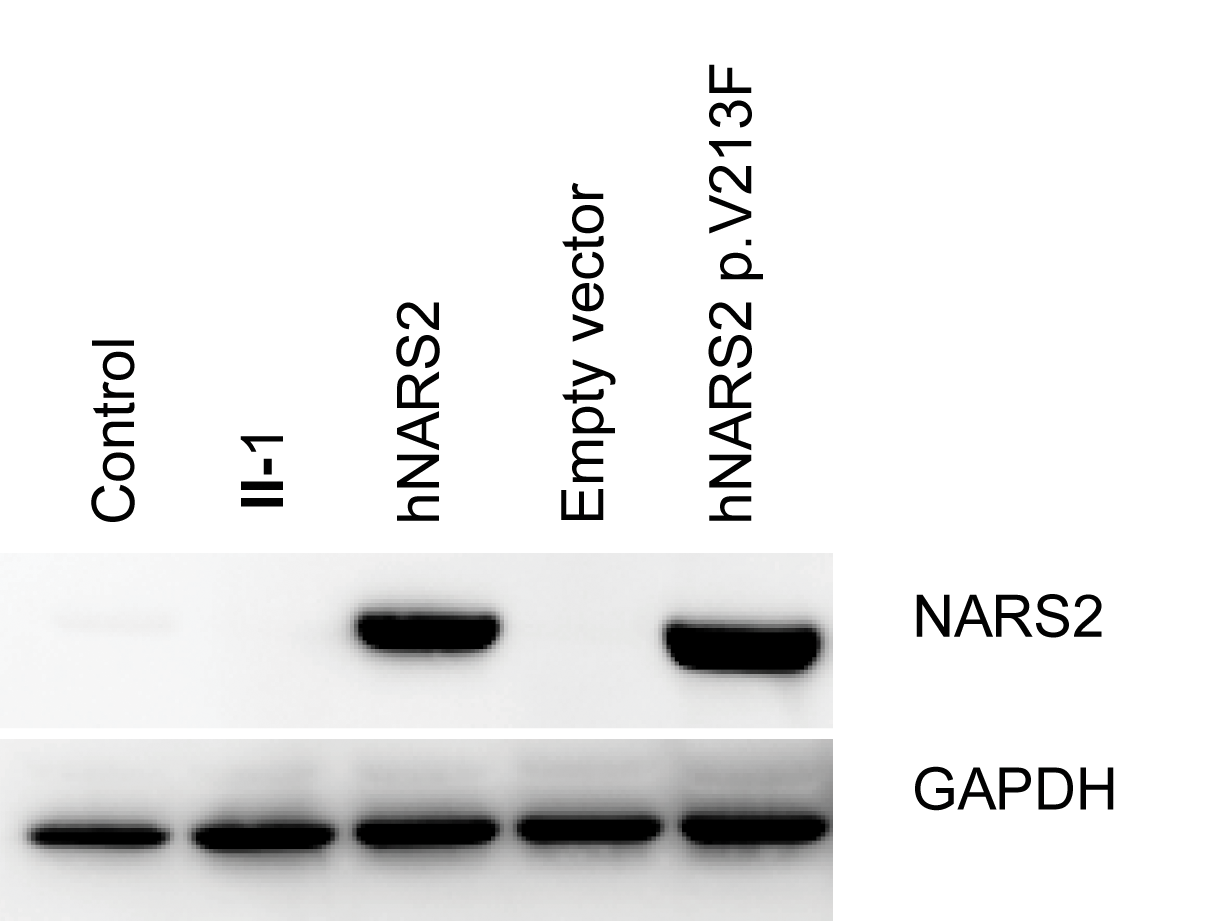

Supplement: S9 Fig — NARS2 lentiviral constructs were made by cloning human NARS2 cDNA into pLVX-IRES-tdTomato vector and then packaged into pseudoviral particles. NARS2 expression was assessed with Western Blot in transduced patient cells to monitor the transduction efficiency, using an anti-NARS2 antibody. GAPDH antibody was used as a loading control. (TIF) [file pgen.1005097.s017.tif]
